# Leveraging ML for profiling lipidomic alterations in breast cancer tissues: a methodological perspective

**DOI:** 10.1038/s41598-024-71439-7

**Published:** 2024-10-28

**Authors:** Parisa Shahnazari, Kaveh Kavousi, Zarrin Minuchehr, Bahram Goliaei, Reza M Salek

**Affiliations:** 1 Laboratory of Complex Biological Systems and Bioinformatics (CBB), Department of Bioinformatics, Institute of Biochemistry and Biophysics (IBB), University of Tehran, Tehran, Iran; 2 Bioinformatics Group, Kish International Campus, University of Tehran, Kish Island, Iran; 3 Department of Systems Biotechnology, National Institute of Genetic Engineering and Biotechnology, Tehran, Iran; 4 Laboratory of Biophysics and Molecular Biology, Institute of Biochemistry and Biophysics (IBB), University of Tehran, Tehran, Iran; 5 School of Clinical Medicine, University of Cambridge, Cambridge Biomedical Campus, Cambridge, CB2 0SP United Kingdom

**Keywords:** Lipidomics, Breast cancer, Feature selection, Machine learning, Univariate analysis, Multivariate analysis, Breast cancer, Predictive markers, Computer modelling, Computational models, Data mining, Machine learning, Computational biology and bioinformatics

## Abstract

In this study, a comprehensive methodology combining machine learning and statistical analysis was employed to investigate alterations in the metabolite profiles, including lipids, of breast cancer tissues and their subtypes. By integrating biological and machine learning feature selection techniques, along with univariate and multivariate analyses, a notable lipid signature was identified in breast cancer tissues. The results revealed elevated levels of saturated and monounsaturated phospholipids in breast cancer tissues, consistent with external validation findings. Additionally, lipidomics analysis in both the original and validation datasets indicated lower levels of most triacylglycerols compared to non-cancerous tissues, suggesting potential alterations in lipid storage and metabolism within cancer cells. Analysis of cancer subtypes revealed that levels of PC 30:0 were relatively reduced in HER2(−) samples that were ER(+) and PR(+) compared to those that were ER(−) and PR(−). Conversely, HER2(+) tumors, which were ER(−) and PR(−), exhibited increased concentrations of PC 30:0. This increase could potentially be linked to the role of Stearoyl-CoA-Desaturase 1 in breast cancer. Comprehensive metabolomic analyses of breast cancer can offer crucial insights into cancer development, aiding in early detection and treatment evaluation of this devastating disease.

## Introduction

Breast cancer is a prevalent life-threatening disease affecting women worldwide and remains one of the leading causes of cancer-related mortality among women^1^. The complex nature of the disease stems from the dysregulation of multiple genes, epigenetic factors, and protein interactions, manifesting as metabolic pathway alternations^2^. Metabolomics, encompassing the analysis of a set of metabolites in biological samples, represents a potent tool for detecting, classifying, and assessing the progression and classification of breast cancer^3^. In cancer studies, metabolites can be collected from various tissues and biofluids such as blood, urine, or saliva^4^. By studying the metabolomics profile of breast cancer tissue and comparing it to the metabolic profile of normal tissue, one can derive insights into the metabolic pathway alternations in cancer cells due to proliferation and metastasis. Metabolomics has the potential to play a significant role in the early detection of cancer, screening, and monitoring treatments^5,6^. Lipidomics identifies and quantifies lipids extracted from biological samples and cancer cells^7,8^. Lipids like phosphatidylcholine and phosphatidylethanolamine play a crucial role in the progress of breast cancer^9^.

To enhance the selection of metabolite profiles and the biological interpretation of selected biomarkers in this study, a focus was placed on saturated and mono-unsaturated phospholipids. Previous studies have shown these phospholipids to be upregulated in breast cancer tissues. Consequently, the comprehensive results obtained, which align with prior findings, clearly demonstrate the robustness of the employed methodology.

In the current study, eight lipid families were extracted from earlier Liquid Chromatography–Mass Spectrometry (LC–MS) data in positive and negative modes from both normal and cancerous breast tissues. The lipid families include phosphatidylcholine (PC), lysophosphatidylcholine (LPC), phosphatidylethanolamine (PE), phosphatidylserine (PS), phosphatidylinositol (PI), ceramide (Cer), sphingomyelin (SM), and triacylglycerol (TAG). Their roles in breast cancer were analyzed following established literature and are highlighted below.

Studies by Bogdanov et al.^10^ and Patel et al.^11^ have shown that PE acts as a mediator and regulator of multiple signaling pathways and functions as a lipid chaperone, facilitating the appropriate folding of membrane proteins. However, excessive accumulation of misfolded proteins can result in chronic endoplasmic reticulum (ER) stress associated with cancer development^12^. Raynor et al.^13^ and Ross et al.^14^ have reported that saturated LPC, a by-product of PC composed of saturated and monounsaturated phospholipids, may elevate the rigidity of the cell membrane, a factor implicated in metastasis.

Phosphatidylinositol is a signaling molecule in eukaryotic cells and a vital component of the cell membrane. It plays a crucial role in regulating cancer cell proliferation, including cell signaling pathways, survival, adhesion, motility, and angiogenesis. Multiple studies have shown that increased levels of certain PIs in breast cancer indicate their potential role in cancer development and progression^15^.

In a study by Tallima et al.^16^, the significance of SM in the cell surface was highlighted. SM, a prevalent sphingolipid in mammalian cell membranes, concentrates predominantly in the outer membrane leaflet. It interacts with cholesterol and proteins, forming rafts in the cell membrane that play a crucial role in signal transduction pathways, including cell adhesion, migration, survival, proliferation, and apoptotic signaling^17^. In particular, high levels of SM, particularly saturated and monounsaturated forms, are linked to the rigidity of raft lipids in malignant cells^18^.

Triacylglycerols play a crucial role as transporters for fatty acids and are the primary source of energy storage in animal fat. They are stored in lipid droplets and can be quickly released when needed. Recent studies by Mika et al. and Eckeret et al. suggested that tumor tissues in colorectal cancer have lower levels of TAGs and higher levels of cell membrane lipids^19,20^.

Reprogramming lipogenesis enhances the cell membrane composition by generating saturated and mono-unsaturated phospholipids^21^. These phospholipids help resist ferroptosis—a form of cell death dependent on iron and characterized by the accumulation of lipid peroxides^22,23^. Previous studies showed breast cancer progression is stimulated by de novo lipogenesis, with increased membrane saturation from saturated and mono-unsaturated phospholipids. This is primarily attributed to SCD1 overexpression, a crucial lipogenic enzyme that supports cancer growth by converting saturated fatty acids into Δ9-monounsaturated fatty acids (MUFAs) such as palmitoleic acid and oleic acid (nonessential fatty acids)^24,25^. SCD1 expression is regulated by multiple hormonal factors, with insulin and growth factors upregulating it, while estrogen and leptin suppress it^26^. Furthermore, both triple-negative (HER2−, ER−, and PR−) and HER2-rich (ER(−), PR(−), and HER2 positive) breast cancer patients have been reported to exhibit overexpression of SCD1^27,28^.

## Results

### Overview of workflow pipeline for ML data analysis

**Fig. 1:**
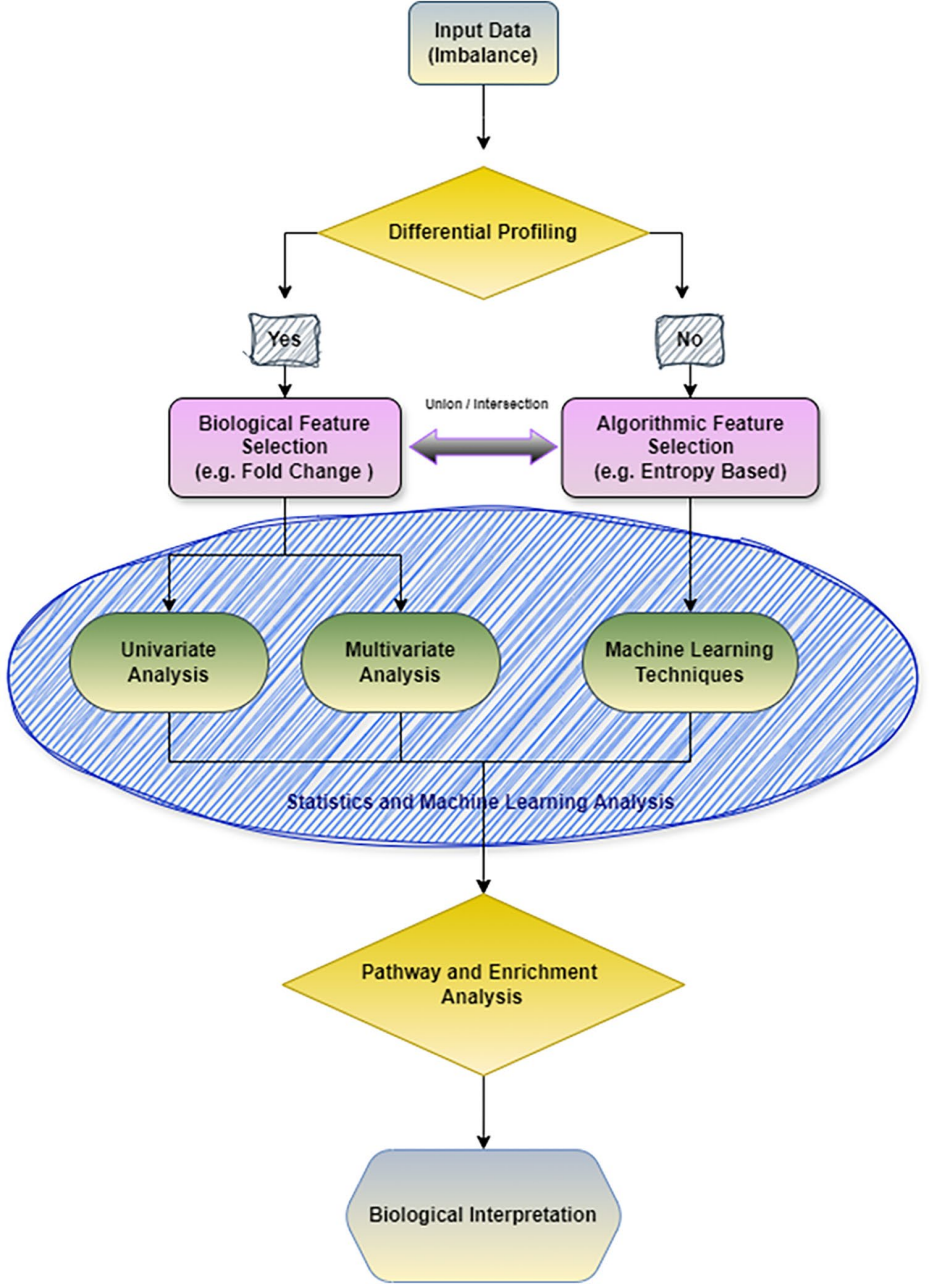
Indicative of metabolic, including lipidomic, dysregulation in breast cancer, the input datasets initially undergo comprehensive preprocessing steps. Algorithmic feature selection methods, including MLP and entropy-based techniques, are utilized alongside fold change and effect size criteria to select features based on predefined thresholds. Subsequent analyses involve univariate, multivariate, and machine learning approaches to identify significant lipids. Finally, pathway and enrichment analyses are conducted to interpret the metabolite profiles within the context of a lipidomic reaction network.

The overall workflow of metabolomics/lipidomics analysis using machine learning to identify significant metabolite profiles is illustrated in Fig. 1. Initially, individual datasets were subjected to normalization processes such as log2 transformation, scaling, and median-centering as necessary. To address any dataset imbalances, oversampling techniques were applied during the machine learning process. Differential profiling using fold change or effect size was performed to assess significant differences between groups of interest. If no significant differences were observed, algorithmic feature selection was employed exclusively to identify significant metabolites or lipids. However, when differential profiling revealed notable differences, the selected features were further analyzed using univariate and multivariate techniques, alongside machine learning classification approaches. To achieve optimal performance, the combination and intersection of biological and algorithmic feature selection methods were considered. The final selected metabolites underwent pathway and enrichment analysis, followed by biological interpretation using relevant databases and literature. This workflow is versatile and can be applied across various metabolomics platforms.

### Feature selection and model optimization

**Fig. 2:**
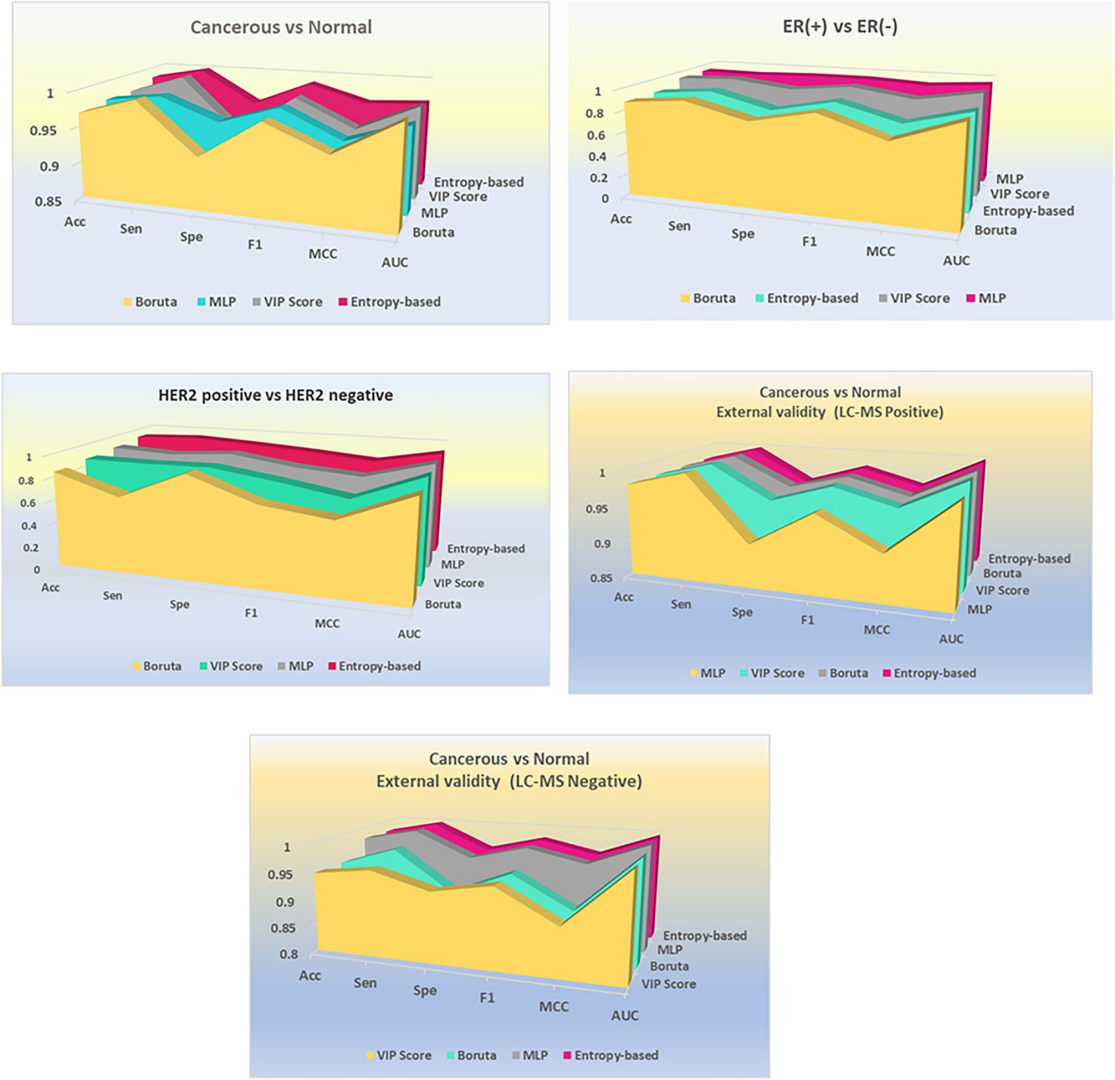
Comparative analysis of feature selection methods. Comparison of four feature selection methods (Boruta, MLP, Entropy-based, and VIP score) using binary logistic regression for original (positive mode) and external validation (both modes) data. The analysis compares Normal vs. Cancerous, ER-positive vs. ER-negative, and HER2-positive vs. HER2-negative for original data, and Normal vs. Cancerous for external validation data. Evaluation metrics include accuracy, sensitivity, specificity, F1 score, MCC, and AUC score. The Entropy-based method performs best in distinguishing cancerous vs. normal status in both original and external validation data, as well as HER2 status. MLP excels in identifying important features between ER-positive and ER-negative class labels.

Four algorithmic feature selection methods, including Boruta, Multilayer Perceptron (MLP), Entropy-based, and Variable Importance in Projection (VIP) score, were applied to the positive mode LC–MS datasets, along with external validation for both modes. Figure 2 presents the results of binary logistic regression used to evaluate the feature selection methods across three groups of interest for original data (METACancer FP7 project) and external validation (project PR000742). For the negative mode LC–MS data, the selected features from the original data using all algorithmic and biological feature selection methods totaled seven lipids, which are not illustrated in the figure.

In this study, a heuristic approach was adopted, wherein the top 10% to 50% of features were retained to determine the optimal number of features. Meanwhile, the optimal feature count varied from the defined range, depending on the dataset and the specific analysis requirements. The range was subsequently refined based on evaluation metrics, ensuring that the final feature selection yielded optimal performance. Initially, 183 features were considered as annotated lipids for the positive mode datasets. After feature selection, the optimal feature numbers for the cancerous vs. normal, Her2, and ER status were found to be 90, 100, and 73, respectively.

Among the feature selection methods, the Entropy-based approach exhibited superior performance compared to the other three methods in classifying cancerous vs. normal and HER2 status class labels, in the original datasets. For the normal vs. cancerous group, the Entropy-based method demonstrated the highest overall values for Accuracy (0.9843), Sensitivity^1^, Specificity (0.9538), F1-score (0.9882), MCC (0.965), and AUC (0.97). Similarly, for the HER2 status, the Entropy-based approach achieved the highest scores for Accuracy (0.9213), Sensitivity (0.9444), F1-score (0.8718), and MCC (0.821). In the context of ER status, the MLP model exhibited the highest scores for Accuracy (0.9387), Sensitivity (0.932), Specificity (0.95), MCC (0.9505), and F1-score (0.921), surpassing other models in accurately identifying the ER-positive group.

The biological feature selection approach, specifically utilizing the median log2 Fold Change method, was employed for both univariate and multivariate analyses of the original datasets to identify features with high discriminatory power. Tables S1 and S2 present phospholipids detected in positive and negative ionization modes. The common lipids identified across both modes include PC 32:0, PC 34:1, PC 34:2, PC 36:1, PE 36:1, PE 38:2, SM 34:1, and SM 36:1. The detection of these lipids in both LC-positive and LC-negative modes confirms the accuracy and reproducibility of lipid measurements, highlighting the biological relevance of these lipids. The final selected saturated and mono-unsaturated phospholipids for both positive and negative LC–MS data are detailed in Table 1. This feature selection process was consistently applied to each specific interest status, including ER, PR, and HER2, with the corresponding outcomes presented in Table 2. Optimal thresholds for median log2 fold changes of ≥ 1.8 and ≤  − 1.5 were identified to distinguish between normal and cancerous groups. Values of ≥|1.2| were selected for the subtype groups. The PR status observed a notable fold change of − 1.50674 with a p-value of 7.91 × 10^–5^ for PC 30:0.

**Table 1:** Differential accumulation of saturated and mono-unsaturated phospholipids (LPC, PC, PE, PI, and SM) in normal and cancerous breast tissues using LC–MS.

No	Lipid name	PubChem-CID	Log2 FC	Effect size	AUC	MCC
LC–MS positive mode						
1	LPC 16:0	460602	1.986	1.899	0.82	**0.481**
2	LPC 18:0	497299	2.436	1.793	0.85	0.666
3	PC 30:0	129657	3.949	2.599	0.94	0.799
4	PC 30:1	24778575	3.453	2.154	0.93	0.790
5	PC 32:0	452110	4.097	3.018	0.94	0.824
6	PC 32:1	53478711	4.580	2.689	0.95	0.819
7	PC 34:1	5497103	4.163	3.310	0.96	0.864
8	PC 36:1	24778825	4.550	3.135	0.97	0.883
9	PC 38:1	24778843	3.179	2.226	0.93	0.722
10	PC 37:1	52922336	3.078	2.040	0.94	0.825
11	PE 34:1	5283496	4.074	2.783	0.94	0.779
12	PE 33:1	52924162	2.472	2.022	0.95	0.767
13	PE 32:1	52994944	4.412	2.775	0.96	0.786
14	PE 36:0	52924864	5.238	2.791	0.96	0.824
15	PE 36:1	9546755	4.841	3.178	0.96	0.824
16	PE 38:1	9546837	4.203	2.658	0.96	0.813
17	PE 39:0	52924482	** − 2.1448**	** − 1.4260**	–	–
18	SM 34:0	9939965	3.563	2.420	0.92	0.734
19	SM 32:1	11433862	2.486	1.769	0.88	0.644
20	SM 33:1	52931137	2.649	1.758	0.87	0.682
21	SM 34:1	9939941	3.686	2.555	0.94	0.786
22	SM 35:1	46891763	3.053	2.1270	0.95	0.773
23	SM 36:1	52931165	2.724	2.0320	0.87	0.695
24	SM 39:1	154573100	**0.564**	**0.7562**	–	–
25	SM 40:1	52931165	2.139	1.771	0.92	0.760
LC–MS negative mode						
1	PC 36:1	52922418	1.931	1.512	0.92	0.733
2	PE 36:1	52922418	2.033	2.135	0.94	0.744
3	PC 32:1	6443788	2.187	2.225	081	0.543
4	PI 36:1	52928410	1.907	1.824	0.90	0.667

The table provides data on the differential accumulation of phospholipids analyzed in positive and negative modes of LC–MS between normal and cancerous breast tissues. Feature selection was based on Entropy-based elimination, median log2 fold change (≥ 1.8 or ≤  − 1.5), effect size Cohen’s d ( ≥|1|), and p-values from adjusted t-tests < 0.01 and nonparametric MWU tests < 0.01. Only features that also met additional criteria, including Matthews correlation coefficient (MCC) ≥ 0.5 and area under the curve (AUC) score ≥ 0.80 from SVM-poly classification, were included in the analysis. The significance level after Bonferroni correction was adjusted to 5.4645e−05 for LC-positive mode and 0.0003 for LC-negative mode All features included in the table were significant according to the Bonferroni-corrected thresholds, and p-value results from t-tests, which were all below these thresholds, are not shown in the table.

The bolded values represent the highest performance metrics compared to the other models.

**Table 2:** Differential accumulation of lipids in ER and HER2 status.

Lipid name	PubChem-CID	Log2 FC	p.value (t-test)	Adjusted p.value (FC)	MWU	Effect size	Adjusted p.value (ES)
ER							
PC 30:0	24778679	− 1.501	****	**	****	− 0.545	***
PC 32:1	24778620	− 1.127	***	**	***	− 0.458	***
PE 34:1	5283496	− 1.026	***	*	***	− 0.450	**
PE 32:1	24778620	− 1.464	****	***	****	− 0.458	***
PR							
PC 30:0	24778679	− 1.507	***	***	***	− 0.624	**
HER2							
PC 30:0	24778679	1.386	**	*	**	0.493	*
PC 30:1	24778615	1.824	***	**	****	0.696	**
PC 32:1	24778620	1.498	***	**	***	0.660	**
SM 34:0	9939965	1.142	*	*	**	0.425	*
SM 34:1	9939941	1.221	**	*	*	0.326	*
SM 32:1	11433862	1.333	**	*	**	0.419	**

Lipid accumulation differences between ER and HER2 groups were assessed using specific criteria. The selection process involved machine learning (ML) feature selection, requiring a fold change of |log FC| ≥ 1.2, an effect size |d| > 0.4, and p-values from t-tests and Mann–Whitney U (MWU) tests both < 0.01. Significance levels are indicated as follows: *p < 0.1, **p < 0.01, ***p < 0.001, with Bonferroni-corrected significance denoted as ****. The Bonferroni correction adjusted the significance thresholds to 0.00125 for HER2, 5.4645e−05 for ER subtypes, and 0.003 for PR subtypes.

### Comparison of SVM and random forest classification

The performance of four machine learning classifiers—SVM-linear, SVM-radial, SVM-Polynomial, and Random Forest—was assessed for predicting tumor and breast cancer subtypes after applying oversampling and feature selection techniques. The evaluation metrics provided in Table 3 outline the effectiveness of these classifiers in distinguishing between normal and malignant tissues. SVM-Polynomial demonstrated superior performance in both positive and negative modes. In LC–MS positive, it accurately identified normal tissues (specificity: 1 ± 0) and showed robust detection of malignant tissues (sensitivity: 0.9538 ± 0.013), achieving an accuracy of 0.9843 ± 0.009. The classifier exhibited a favorable balance between precision and recall (F1 score: 0.9764 ± 0.007), along with good discriminative ability (AUC-ROC: 0.97) and a strong correlation between predicted and actual classes (MCC: 0.965). Similarly, in the LC–MS negative, SVM-Polynomial achieved high accuracy (0.9911 ± 0.009) and demonstrated precise identification of normal tissues (specificity: 1 ± 0) and effective detection of malignant tissues (sensitivity: 0.9643 ± 0.035). The models also exhibited a favorable F1 score (0.9818 ± 0.096), excellent discriminative ability (AUC-ROC: 1), and a strong correlation (MCC: 0.976) between predicted and actual classes. Table 4 presents the evaluation metrics of the defined classifiers for predicting the ER and HER2 status. In terms of ER classification, SVM-Poly achieved the highest accuracy (0.9693 ± 0.014), specificity (0.9508 ± 0.028), sensitivity (0.9804 ± 0.014), and F1 score (0.9654 ± 0.134). The models demonstrated a favorable AUC-ROC of 0.99 and an MCC of 0.934. For HER2 classification, SVM-Radial achieved the highest accuracy (0.9528 ± 0.019), sensitivity (1 ± 0), specificity (0.9381 ± 0.024), and F1 score (0.9681 ± 0.128), along with an AUC of 0.99.

**Table 3:** Evaluation metrics for classification in positive and negative modes following entropy-based feature selection between cancerous and normal tissues.

	SVM-linear	SVM-radial	SVM-poly	Random Forest
LC–MS positive				
Accuracy	0.9738 ± 0.012	0.9738 ± 0.012	**0.9843 ± 0.009**	0.963 ± 0.014
Specificity	0.9841 ± 0.013	1 ± 0	**1 ± 0**	1 ± 0
Sensitivity	0.9538 ± 0.022	0.9231 ± 0.017	**0.9538 ± 0.013**	0.947 ± 0.019
F1	0.9612 ± 0.009	0.9600 ± 0.018	**0.9764 ± 0.007**	0.973 ± 0.01
AUC-ROC	0.97	0.98	**0.97**	0.98
MCC	0.942	0.942	**0.965**	0.930
LC–MS negative				
Accuracy	0.9643 ± 0.018	0.9643 ± 0.018	**0.9911 ± 0.009**	0.9554 ± 0.02
Specificity	0.96 ± 0.039	0.9765 ± 0.016	**1 ± 0**	0.9878 ± 0.012
Sensitivity	0.9655 ± 0.02	0.9259 ± 0.05	**0.9643 ± 0.035**	0.8667 ± 0.062
F1	0.9767 ± 0.011	0.9505 ± 0.161	**0.9818 ± 0.096**	0.9123 ± 0.203
AUC-ROC	0.97	0.99	**1**	0.99
MCC	0.926	0.872	**0.976**	0.906

Classification metrics for SVM (Linear, Radial, and Polynomial kernels) and Random Forest are reported after entropy-based feature selection. Standard deviation (STD) values are included for each metric.

The bolded values represent the highest performance metrics compared to the other models.

**Table 4:** Classification evaluation metrics for ER and HER2 status with feature selection using SVM (linear, radial, and polynomial kernels) and Random Forest.

	SVM-linear	SVM-radial	SVM-poly	Random Forest
ER				
Accuracy	0.9387 ± 0.019	0.9264 ± 0.02	**0.9693 ± 0.014**	0.9387 ± 0.019
Specificity	0.931 ± 0.033	0.9138 ± 0.037	**0.9508 ± 0.028**	1 ± 0
Sensitivity	0.9429 ± 0.023	0.9333 ± 0.024	**0.9804 ± 0.014**	0.9115 ± 0.027
F1 score	0.9369 ± 0.183	0.9235 ± 0.204	**0.9654 ± 0.134**	0.9537 ± 0.156
AUC-ROC	0.95	096	**0.99**	0.98
MCC	0.848	0.843	**0.934**	0.897
HER2				
Accuracy	0.9449 ± 0.02	**0.9528 ± 0.019**	0.9213 ± 0.024	0.9134 ± 0.025
Specificity	0.9667 ± 0.019	0.9381 ± 0.024	0.9355 ± 0.025	0.9082 ± 0.029
Sensitivity	0.8919 ± 0.051	**1 ± 0**	0.8824 ± 0.055	0.931 ± 0.047
F1 score	0.9278 ± 0.197	**0.9681 ± 0.128**	0.9081 ± 0.225	0.9195 ± 0.209
AUC-ROC	0.96	**0.99**	0.95	0.96
MCC	0.852	**0.864**	0.848	0.802

Standard deviation (STD) values are provided for each metric.

The bolded values represent the highest performance metrics compared to the other models.

The superior performance of these classifiers, particularly SVM-Polynomial, underscores their efficacy in selecting significant lipids, as indicated by high AUC and MCC values.

### Distinguishing breast cancer tissues and subtypes

Prior publications^9,11,23,29^ and our preliminary analysis of positive mode of LC–MS data showed that lipid levels in cancerous tissues surpass those observed in normal counterparts. The quantitative analysis of lipid signal intensities (excluding triacylglycerols) in malignant tumors demonstrated a remarkable 29.8-fold higher concentration than in normal tissue. Specifically, cancerous tissues exhibited a significantly higher accumulation of saturated and monounsaturated phospholipids, a 23.48-fold increase compared to normal cells.

#### Univariate and multivariate approaches: cancerous vs. normal tissues

**Fig. 3:**
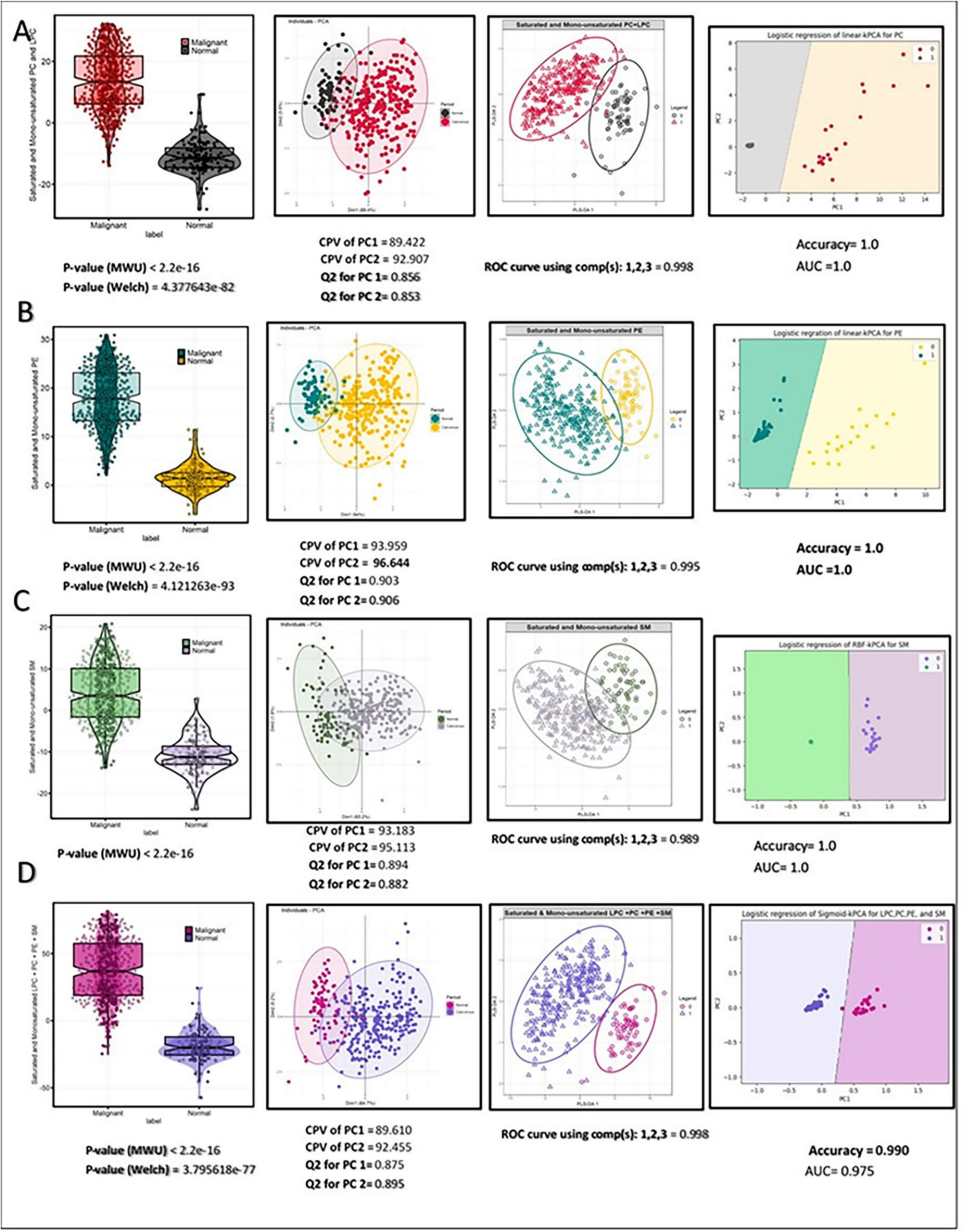
The differential accumulation of selected lipids was analyzed using both univariate and multivariate approaches in positive mode. The differential accumulation of selected lipids was analyzed using both univariate and multivariate approaches in positive mode. Data distribution was assessed with the Shapiro–Wilk test (p-value < 0.05) to determine normality. For data not following a normal distribution, the Welch correction was excluded. The distinction between cancerous and normal tissues was illustrated through PCA and PLS-DA score plots, displaying eigenvalues and cumulative percent variance (CPV) of PC1 and PC2 in PCA. PLS-DA evaluation involved the use of AUC-ROC curves, averaging ROC curves from components 1, 2, and 3. (**a**) Linear Kernel PCA (kPCA) with logistic regression was used to analyze the differential abundance of saturated and monounsaturated PC and LPC. (**b**) Linear-kPCA was employed to identify the optimal parameter for saturated and monounsaturated PE. (**c**) RBF-kPCA (Radial Basis Function-kPCA) was determined as the optimal parameter for saturated and monounsaturated SM. (**d**) Sigmoid-kPCA was applied to analyze saturated and monounsaturated LPC, PC, PE, and SM.

The accumulation of saturated and monounsaturated phospholipids in cancerous compared to normal breast tissues in both polarity modes is shown in Table 1. The lipids with the highest variation—LPC, PC, PE, PI, and SM—were selected using an entropy-based feature selection method. The criteria for inclusion of these lipids were as follows: a median log2-fold change of ≥ 1.8 or ≤  − 1.5, an effect size (Cohen’s d) of ≥ |1|, a Mann–Whitney U test (MWU) p-value of ≤ 0.01, an adjusted t-test p-value of ≤ 0.01 using the Benjamini–Hochberg (BH) method, and an MCC of ≥ 0.5. For lipids that were normally distributed, the Welch t-test was also considered. Additionally, the significance level for each feature was adjusted using the Bonferroni correction, resulting in a Bonferroni-corrected significance level of 6.061e−05 for LC-positive lipids and 0.0003 for LC-negative lipids. The SVM-polynomial algorithm was chosen for modeling, as it demonstrated an AUC greater than 0.8. Table S2 represents phospholipids in LC-positive original data. The statistical models, which assess the differential abundance of saturated and monounsaturated selected phospholipids between cancerous and normal patients in LC–MS positive mode, are presented in Fig. 3. The combination of fold change and entropy-based feature selection methods was used to differentiate between cancerous and normal status. The bar plots comparing saturated and mono-unsaturated LPC, PC, PE, and SM between cancerous and normal tissues reveal a significantly higher abundance of these lipids in the cancerous samples. Figure 3A presents a violin plot comparing the medians of saturated and monounsaturated phosphatidylcholine and lysophosphatidylcholine (LPC 18:0, PC 30:0, PC 30:1, PC 32:0, PC 32:1, PC 34:1, PC 36:1, PC 38:1, PC 37:1). Statistical tests, including Welch’s t-test (p-value: 4.377643e−82) and the MWU test (p-value: < 2.2e−16), were conducted to assess the significance of the observations. The results indicated a statistically significant difference (p < 0.01) in medians between the two tissue types.

Principal Component Analysis (PCA) distinguished normal and cancerous tissues with CPV values of 89.422 for PC1 and 92.907 for PC2. High Q2 values of 0.856 for PC1 and 0.853 for PC2 indicated meaningful discrimination between the tissue types. Subsequently, Partial Least Squares Discriminant Analysis (PLS-DA) revealed a significant distinction between cancerous and normal samples. The average AUC for components 1, 2, and 3 was 0.998, highlighting a robust ability to differentiate between the two tissue types. Figure 3B illustrates a group bar plot comparing the levels of saturated and mono-unsaturated phosphatidylethanolamine (PE 34:1, PE 33:1, PE 32:1, PE 36:0, PE 36:1, and PE 38:1) in positive mode between cancerous and normal tissues. The results of the Welch and MWU tests showed significant differences between the two groups, with p-values of 4.12e−93 and less than 2.2e−16, respectively. The PCA analysis performed on PEs demonstrates a distinct separation between the two groups, as indicated by CPV values of 93.959 for PC1 and 96.644 for PC2. Additionally, the compelling predictive performance is evident from the Q^2^ values of 0.903 for PC1 and 0.906 for PC2, respectively, further confirming the accuracy of the predictive model. Moreover, the ROC curve analysis using PLS-DA components revealed an AUC of 0.995, further validating the analysis’s robustness.

Further analysis using linear-kernel PCA on saturated and monounsaturated PC and PE showed a clear, distinct difference between malignant breast tumors and normal tissues, as seen in Fig. 3A,B. This approach was further supported by the logistic regression analysis, which demonstrated complete separation between the two groups and achieved perfect accuracy and AUC scores (both equaling 1). Figure 3C shows the violin plot comparing saturated and mono-unsaturated sphingomyelin (SM 34:0, SM 32:1, SM 33:1, SM 34:1, SM 35:1, SM 36:1, and SM 40:1) between malignant and normal tissues. The distinction between malignant and normal tissues was also achieved using PCA, with CPV and Q^2^ values of 95.113 and 0.894, respectively, and PLS-DA, with an average ROC curve of 0.989. The RBF-kPCA analysis of saturated and monounsaturated SMs also distinguished malignant breast tumors and their matched normal tissues.

The difference between saturated and mono-unsaturated PC, LPC, PE, and SM can be seen in Fig. 3D. The results of the violin plots, which used MWU and Welch tests, indicate a significant difference in expression between cancerous and normal samples. The p-values were > 2.2e−16 and 3.795618e−77, respectively. PCA and PLS-DA analysis showed a clear separation between the two groups, with PLS-DA achieving an AUC curve of 0.998. Sigmoid-kernel PCA was employed after fine-tuning through threefold cross-validation to enhance classification accuracy. The best kernel resulted in an accuracy of 0.990 and an AUC score of 0.975.

The difference in PC, PE, and PI levels in negative mode between normal and malignant breast tissue samples is shown in Fig. S1 and Table S2. Notably, a significant up-regulation was observed in cancerous breast tissues for PE 40:6, PE 36:3, PI 36:1, PC 36:1, and PE 36:1.

#### Univariate and multivariate approaches: breast cancer subtypes

**Fig. 4:**
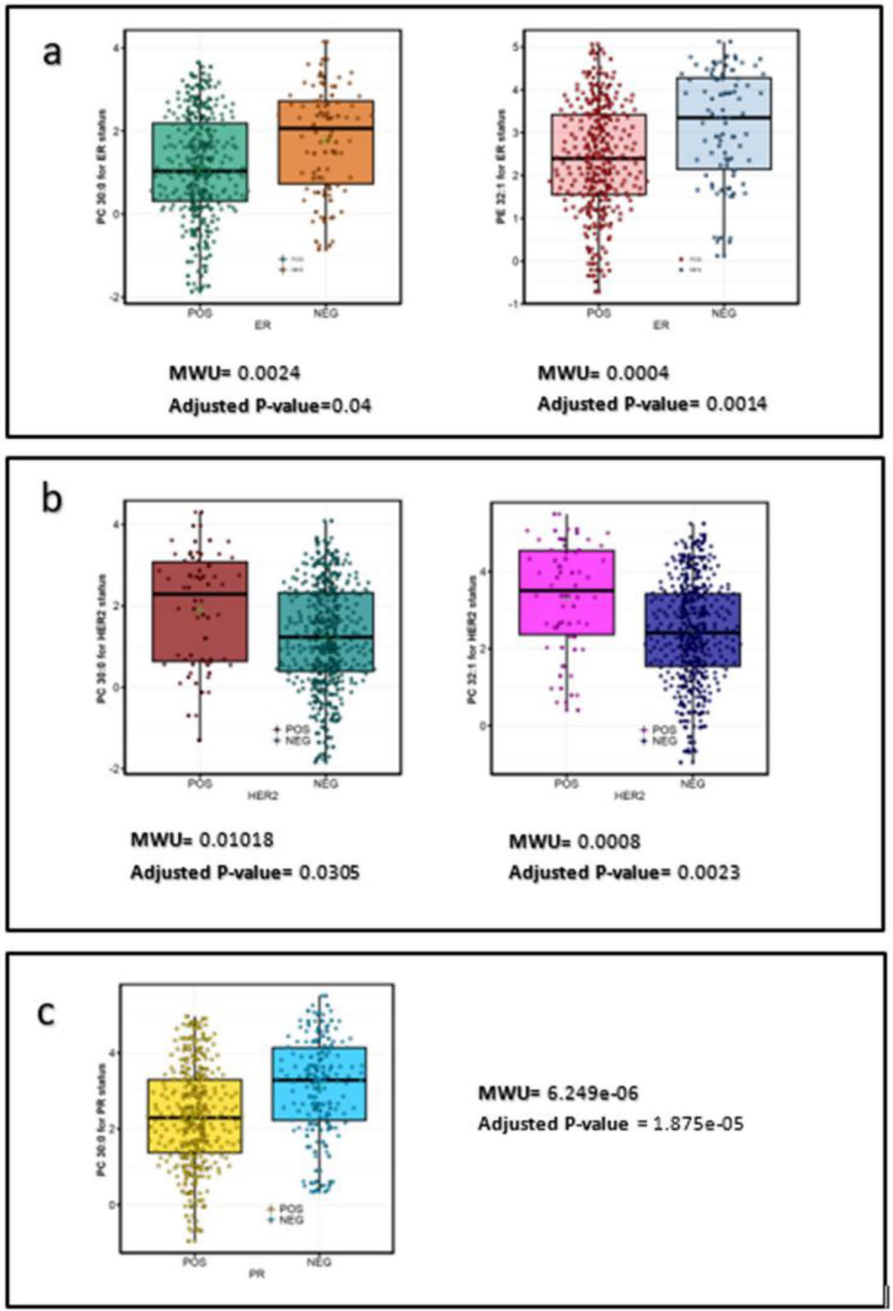
Differential abundance analysis of selected saturated and mono-unsaturated phospholipids based on ER, HER2, and PR Status. (**a**) Downregulation of PC 30:0 and PE 32:1 in ER+ samples. (**b**) Upregulation of PC 30:0 and PC 32:1 in HER2(+) samples. (**c**) Downregulation of PC 30:0 in PR-positive (PR+) breast cancer tissues. For lipids with non-normal distribution, Welch’s correction was excluded. Normality was determined using the Shapiro–Wilk test (p-value < 0.05). A Bonferroni correction was applied to adjust the significance levels to 5.46e−05, 0.00125, and 1.88e−05 for ER, HER2, and PR subtypes, respectively.

The selection of significant lipids in hormone receptors and HER2 status was conducted by intersecting defined machine learning and biological feature selection methods, along with the previously mentioned assessment criteria. For HER2 status, 94 features were selected using MLP from a total of 183 lipids without prior feature selection, while 5 features were identified using fold change. Regarding ER status, 93 features were chosen using entropy-based feature selection from the original dataset of 183 features, and 5 lipids were identified using fold change. The selection of saturated and mono-unsaturated phospholipids in breast cancer subtypes was based on the criteria presented in Table 2. Notably, PC 30:0 was commonly observed across ER, PR, and HER2 statuses. Furthermore, Fig. 4 presents the results of the differential lipidomics analysis of saturated and monounsaturated phosphatidylcholine (PC) between ER (+) (Estrogen Receptor-Positive, indicating high levels of estrogen in the cancer cells) and ER (−) samples, as well as PR (+) (Progesterone Receptor Positive, indicating high levels of progesterone in the cancer cells) and PR (−) samples, under conditions where HER2 is negative. Additionally, the analysis considers HER2 (+) (Human Epidermal Growth Factor Receptor 2 Positive, indicating high levels of HER2 protein in the cancer cells) and HER2 (−) samples when both ER and PR are negative. The analysis revealed a down-regulation of PC 30:0 and PC 32:1 in ER (+) samples and a down-regulation of PC 30:0 in PR (+) samples. In contrast, HER2 (+) samples showed up-regulation of both PC 30:0 and PC 32:1 in HER2-positive breast cancer tissues.

#### Triacylglycerol levels in cancerous vs. normal tissues

**Fig. 5:**
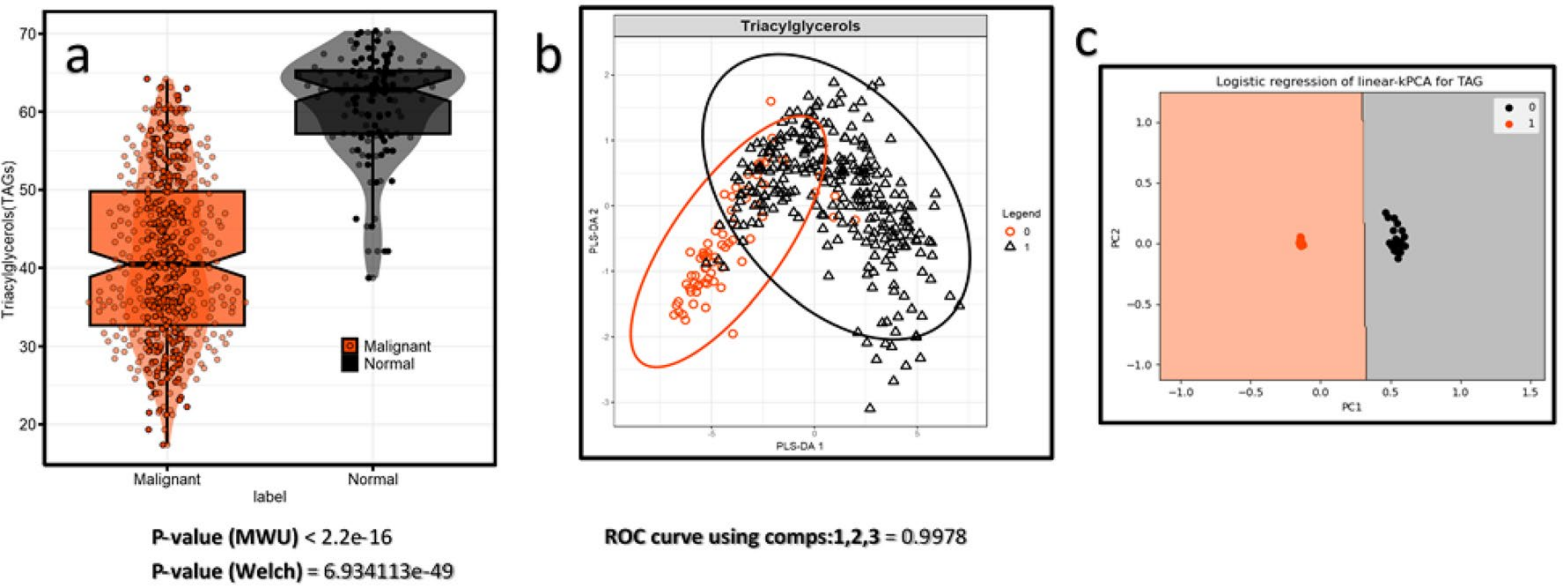
Differential abundance analysis of triacylglycerols (TAGs). (**a**) Differential analysis of TAGs (TAG_42_1, TAG_44_2, TAG_46_2, TAG_48_3, TAG_49_1, TAG_50_4, TAG_50_5, TAG_51_1, TAG_51_2, TAG_52_5, TAG_53_4, TAG_54_6, and TAG_54_7) between cancer and normal samples in positive mode, computed using the defined criteria (MWU and Welch tests). (**b**) PLS-DA score plots with average AUC from PC1, PC2, and PC3. (**c**) Linear-kernel PCA between normal and breast cancer samples. Logistic regression indicates complete segregation between the groups without misclassification.

Table 5 presents a comparative analysis of the differential accumulation of triacylglycerols in the positive mode of LC–MS between malignant and normal cells. The selected TAGs, including TAG 42:1, TAG 44:2, TAG 46:2, TAG 48:3, TAG 49:1, TAG 50:4, TAG 50:5, TAG 51:1, TAG 51:2, TAG 52:5, TAG 53:4, TAG 54:6, TAG 54:7, and TAG 56:8, demonstrated significant downregulation in malignant cells when compared to normal tissues (Fig. 5A). The MWU test showed a p-value < 2.2e−16, indicating strong statistical significance with MCC ≥ 0.5 and ROC AUC curve ≥ 0.8.

**Table 5:** Differential accumulation of selected TAGs in original positive mode data between normal and cancerous samples.

No	Lipid name	PubChem-CID	Log2 FC	Effect size	AUC	MCC
1	TAG 42:1	56936439	− 2.073	− 1.576	0.83	0.552
2	TAG 44:2	56936448	− 1.817	− 1.553	0.82	0.514
3	TAG 46:2	56936466	− 1.571	− 1.548	0.86	0.576
4	TAG 48:3	56936516	− 1.684	− 1.560	0.86	0.583
5	TAG 49:1	56936678	− 1.755	− 1.610	0.85	0.604
6	TAG 50:4	25240359	− 1.802	− 1.651	0.87	0.604
7	TAG 50:5	56937262	− 1.848	− 2.414	0.88	0.594
8	TAG 51:1	56936775	− 2.04	− 1.409	0.82	0.563
9	TAG 51:2	56936536	− 1.555	− 1.391	0.85	0.546
10	TAG 52:5	56937304	− 1.764	− 1.627	0.86	0.659
11	TAG 53:4	56938920	− 1.849	− 1.684	0.84	0.538
12	TAG 53:6	9544242	2.823	1.603	**0.79**	**0.493**
13	TAG 54:6	5322095	− 1.727	− 1.527	0.82	0.557
14	TAG 54:7	348,276,688	− 1.913	− 1.842	0.86	0.576
15	TAG 56:8	56,939,941	− 1.705	− 1.286	**0.79**	0.529

The feature selection process involved entropy-based elimination, median log2 fold change (≥ 1.8 or ≤ − 1.5), effect size Cohen’s d (≥|1|), and p-values from t-tests and nonparametric Mann–Whitney U (MWU) tests both < 0.01. Only features with a Matthews correlation coefficient (MCC) ≥ 0.5 and area under the receiver operating characteristic curve (AUC-ROC) ≥ 0.80 from SVM-poly classification were included in the analysis. The significance level was adjusted to 5.4645e−05 after the Bonferroni correction. p-value results from t-tests, which were all below these thresholds, are not shown in the table.

The bolded values represent the highest performance metrics compared to the other models.

The distribution of TAGs in normal and tumor breast cells was shown using a PLS-DA score plot with an average AUC of 0.997 (Fig. 5B), and linear kernel PCA was employed (Figs. 5C) to highlight the complete separation between normal and cancerous labels in the logistic regression.

Despite an overall decrease in triacylglycerol levels, specific TAG species were upregulated in cancer cells. Notably, TAG 55:6, TAG 57:2, TAG 57:5, TAG 58:5, TAG 58:6, TAG 60:7, and TAG 53:6 showed significant increases (Table S3). This upregulation can be attributed to the cancer cells’ need for particular fatty acids that play crucial roles in cell signaling, membrane fluidity, and interactions with the tumor microenvironment.

### External validation

The same methodology was employed to assess the external validation of saturated and mono-unsaturated phospholipids in Triple Negative Breast Cancer (TNBC) and non-cancerous tissue samples, using entropy-based feature selection for both positive and negative modes (Table S1). The number of features in LC-positive mode was reduced from 117 to 90, while in LC-negative mode, the number of features decreased from 216 to 54. For differential abundance analysis, a threshold of an effect size greater than one was chosen, considering its limited impact on sample size. Table 6 outlines the key features identified through the differential analysis process for LC–MS positive mode. Within the significant features, PC (32:1), PC (36:1), PC (37:1), and PC (38:1) mirrored the original data, exhibiting effect sizes of 1.5848, 1.2974, 2.2003, and 1.8181, respectively. The phospholipid PC (29:1) exhibited remarkable performance in the TNBC versus Normal external validity dataset, showing an effect size of 2.1086, a fold change of 3.9834, an AUC of 0.893, and an MCC of 0.98. Despite these impressive metrics, PC (29:1) is relatively uncommon, and studies specifically examining its role in breast cancer are limited. Conversely, PC (30:1) is more prevalent and potentially more relevant to breast cancer. There is a hypothesis that PC (29:1) might be PC (30:1) due to possible mis-annotation. However, re-annotation of the external data was not feasible due to the absence of retention time and mass-to-charge ratio (m/z) data.

**Table 6:**
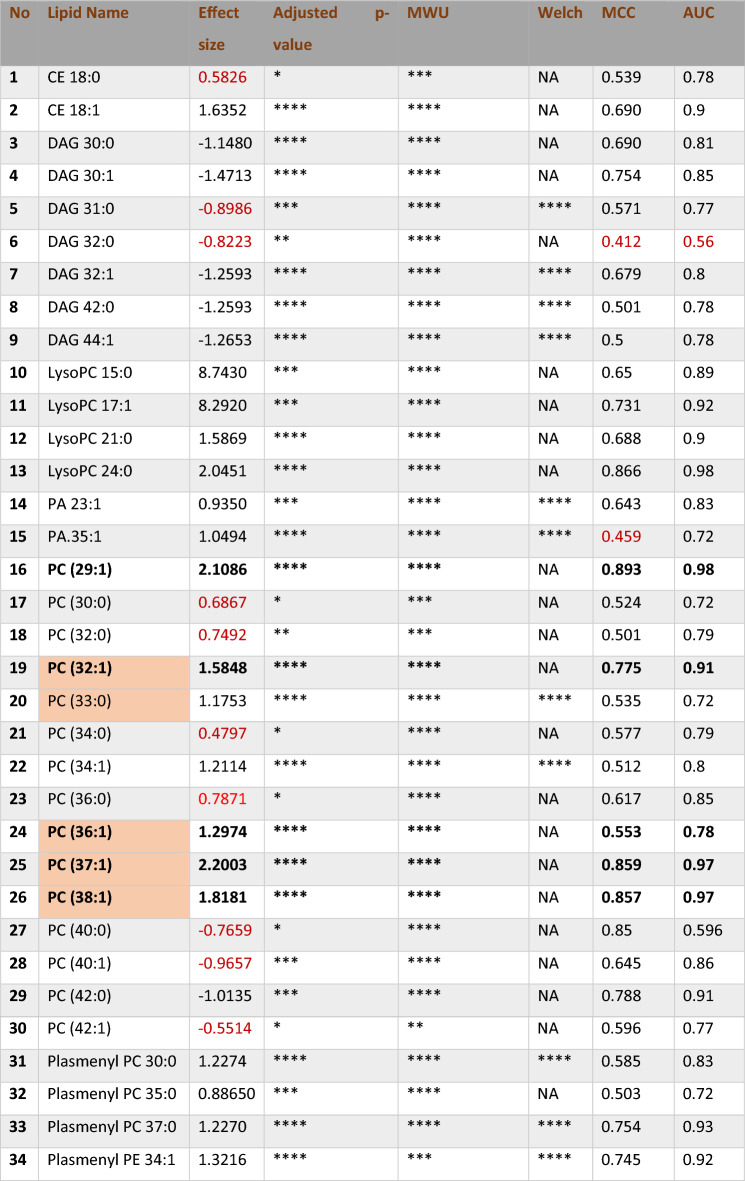
Differential accumulation of selected phospholipids in external validation of LC–MS positive mode.

The phospholipids PC 32:1, PC 33:0, PC 36:1, PC 37:1, and PC 38:1, highlighted in the table, correspond with those identified in the original dataset. The Shapiro–Wilk test was used to assess the normality of the data distribution. Welch’s t-tests (p < 0.01) were applied to normally distributed data, while non-normally distributed lipids were excluded from Welch’s t-tests and marked as NA. Significance levels are indicated as follows: *p < 0.1, **p < 0.01, ***p < 0.001, with Bonferroni-corrected significance, denoted as ****. The Bonferroni correction adjusted the significance threshold to 4.5872e−05.

Figure S2 presents differential comparisons, univariate and multivariate analyses, including a bar plot, PCA, PLSDA, distance separation groups in PLS-DA, and KPCA in LC-positive mode in external validation. The bar plot reveals differential abundance, indicating over-expression of saturated and mono-unsaturated phospholipids in cancerous samples compared to non-cancerous ones. Meanwhile, the segregation between TNBC and Non-cancerous groups is not as distinct as observed in the original data, evidenced by CPV values of PC1 and PC2 (54.148 and 68.776, respectively), alongside an AUC of 0.9741 for PLS-DA. K-PCA demonstrates an AUC of 0.9285 and an accuracy of 0.94 in its logistic regression classification. In LC–MS Negative mode, significant lipids are summarized in Table 7. Only PE (32:1) and PS (36:0) met the defined threshold criteria, with effect sizes of 1.7672 and 1.6774, fold changes of 1.8498 and 2.2077, and AUC values of 0.964 and 0.786 from SVM-Linear, respectively. Acceptability is further supported by MCC values of 0.91 and 0.88.

**Table 7:**
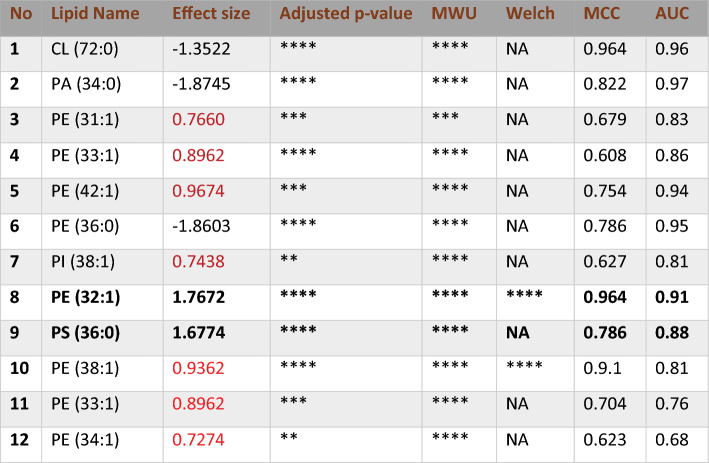
Key lipid signatures identified in LC-NEG for the validation dataset.

Notable phospholipids identified in LC-NEG, including PE (32:1) and PS (30:0) (phosphatidylserine), were selected based on entropy and an effect size of Cohen’s d (≥|1|). These lipids were distinguished by their substantial effect size, MCC, and AUC. All t-tests were conducted similarly in the LC-pos external validation. Significance levels are indicated as follows: *p < 0.1, **p < 0.01, ***p < 0.001. Bonferroni-corrected significance is denoted as ****, with the Bonferroni correction adjusting the significance threshold to 0.0002.

The evaluation metrics for SVMs and RF classifications in both LC–MS modes are detailed in Table 8. SVM-Linear in positive mode and Random Forest in negative mode demonstrated superior performance compared to other classification methods with an accuracy of 0.9821, specificity of 0.9655, sensitivity of 1, F1 score of 0.9825, AUC of 1, and MCC of 1. In the multivariate analysis of LC-Negative data, k-PCA exhibited better segregation than LC-Positive, with an AUC curve and accuracy values of 97.73 and 97, respectively. PLS-DA represented an AUC of 0.9495 (Fig. S3).

**Table 8:** Evaluation metrics for support vector machines (SVMs) and Random Forest classification in positive and negative modes in validation data.

	SVM-linear	SVM-radial	SVM-poly	Random Forest
LC–MS positive				
Accuracy	**0.9821 ± 0.018**	0.9821 ± 0.018	0.9286 ± 0.034	0.9464 ± 0.03
Specificity	**0.9655 ± 0.034**	0.9655 ± 0.034	0.9615 ± 0.038	0.963 ± 0.036
Sensitivity	**1 ± 0**	1 ± 0	0.9 ± 0.055	0.931 ± 0.047
F1	**0.9825 ± 0.094**	0.9825 ± 0.094	0.9298 ± 0.194	0.9467 ± 0.168
AUC-ROC	**1**	1	0.96	0.99
MCC	**1 ± 0**	0.965	0.866	0.965
LC–MS negative				
Accuracy	0.9643 ± 0.025	0.9643 ± 0.025	0.9643 ± 0.025	**0.9821 ± 0.018**
Specificity	0.9333 ± 0.046	0.9333 ± 0.046	0.9333 ± 0.046	**0.9655 ± 0.034**
Sensitivity	1 ± 0	1 ± 0	1 ± 0	**1 ± 0**
F1	0.9655 ± 0.134	0.9655 ± 0.134	0.9655 ± 0.134	**0.9825 ± 0.094**
AUC-ROC	0.98	1	0.97	**1**
MCC	0.931	0.965	0.931	**1**

Feature elimination was conducted using an entropy-based feature selection approach. In LC–MS positive, SVM-linear and SVM-radial exhibited higher performance, while in LC–MS negative, Random Forest demonstrated superior performance.

The bolded values represent the highest performance metrics compared to the other models.

Figure S4 demonstrates the down-regulation of TAGs in the external validation dataset. Figure S8 compares TAG abundance patterns between the original and external validation datasets, revealing consistent trends across numerous triacylglycerols. Although the majority of TAGs exhibit decreased levels, a few show increased levels.

### Pathway and enrichment analysis

Lipids, classified within the Metscape3^30^ framework, encompass several key pathways that play crucial roles in lipid metabolism in breast cancer. These pathways include Arachidonic Acid Metabolism, Glycerophospholipid Metabolism (PC, PE, and PI), Glycosphingolipid Metabolism, Phosphatidylinositol Phosphate Metabolism, and De Novo Fatty Acid Biosynthesis facilitated by SCD1, contributing to lipid metabolism and cellular processes.

The interconnection between the selected structural lipids, including PC, LPC, PE, SM, and PI, is signified as compound–compound interactions within the MetaMapp metabolite network, as shown in Fig. S5a. The size and color of the nodes indicate the magnitude of fold-change, with all selected phospholipids exhibiting upregulation, with a fold-change ranging from 1.9 to 4.7. The compounds are connected using the KEGG reaction pair (krp) and Tanimoto chemical similarity (tmsim). Figure S5b illustrates PC (30:1) conversions and phospholipid relationships within the highlighted key pathways. The enrichment plot in Fig. S6 illustrates the effect sizes for saturated and mono-unsaturated phospholipids in LC-positive samples, comparing cancerous to normal tissues, as well as ER and HER2 statuses. The plots reveal significant up-regulation of selected lipids in cancerous versus normal tissues and in the HER2 subtype, alongside down-regulation in ER status, based on both univariate and multivariate analyses. Figure S7 presents the significant enrichment plot of effect sizes for external validation in LC-positive samples. In Fig. S7b, overlapping lipids in external validation and original data of positive mode are indicated with light green bars. Moreover, plasmalogens, featuring common components such as C16/C18 fatty acids in their saturated or mono-unsaturated forms, are identified as overlapping lipids with the original dataset. These plasmalogens are key components in saturated and mono-unsaturated phospholipids in breast cancer.

## Discussion

This study examined the effectiveness of feature selection methods in analyzing specific lipid groups extracted from LC–MS lipidomics data by comparing four different techniques using binary logistic regression. The entropy-based method outperformed other approaches for the cancerous vs. normal and for HER2 status, whereas MLP demonstrated better performance for ER status. The AUC and MCC scores from the top-performing classification model were used as our criteria to assess individual features. In classification, the SVM-polynomial model exhibited the best performance in both modes of LC–MS for distinguishing between cancerous and non-cancerous samples, as well as for the ER groups. However, for the HER2 subtype, the SVM-radial model demonstrated the highest performance. An ML-assisted workflow, incorporating both univariate and multivariate data analysis, was established to systematically analyze the omics data, with a particular focus on metabolomics, including lipidomics. For optimal feature elimination and selecting significant metabolites, biological feature selection criteria, including fold change, effect size as well and algorithmic feature elimination applied. Using binary machine learning classification techniques for individual feature-selected approaches alongside the combination or intersection of selected features algorithmic and biological feature selection are compared using cross-validation and comprehensive performance metrics to select significant metabolites/lipids. Leveraging machine learning techniques for univariate and multivariate feature selection and classification enhances the robustness of identifying significant lipids and metabolites as metabolite signatures. This workflow thoroughly evaluates and prioritizes features based on their predictive power, ensuring the selected biomarkers are both statistically significant and biologically relevant, thereby improving the accuracy and reliability of biomarker/metabolite profiling in cancer. In addition, a range of statistical methods and measures were employed to evaluate the lipid profile across different breast cancer subtypes, including effect size, median log2 fold change, and the p-values derived from all t-tests.

Enhanced models were derived through feature reduction techniques, resulting in the selection of significant lipids and producing results consistent with those previously reported in the literature. The Bonferroni correction, implemented to adjust the significance threshold for each lipid test individually, was utilized to minimize the likelihood of false positive errors across the entire set of lipid tests. A notable increase in saturated and mono-unsaturated phospholipids levels was observed in LPC, PC, and PE in both positive and negative modes^13,31^. This elevation in fatty acid levels has been associated with heightened tumor cell membrane rigidity, offering protection against oxidative damage and impeding the uptake of chemotherapeutic drugs, thus contributing to cancer progression and infiltration. The results revealed significant involvement of phospholipids, such as PC 30:0, PC 30:1, PC 32:0, PC 32:1, PC 34:1, PC 36:1, PC 38:1, PC 37:1, PE 34:1, PE 33:1, PE 32:1, PE 36:0, PE 36:1, and PE 38:1, in breast cancer development. Moreover, overexpression of sphingomyelins was observed in cancerous tissues, consistent with previous research, emphasizing the involvement of sphingomyelins in cancer reprogramming^18,32^. Specifically, sphingomyelins such as SM 34:0, SM 34:1, and SM 40:1 exhibited high AUC and MCC scores, indicating a high statistical confidence in the role played by sphingomyelin dysregulation in breast cancer.

PCA, PLS-DA score plots, and kPCA were employed to visually represent the distinct changes in saturated and mono-unsaturated lipids (LPC, PC, PE, and SM) between normal and cancerous tissues. The high AUC scores observed for PLS-DA and, notably, kPCA-LR plots of fatty acids confirmed the univariate results. They emphasized the significance of the selected saturated and mono-unsaturated lipids in breast cancer development. Furthermore, the differential abundance of breast cancer subtypes ER, PR, and HER2 was examined (Fig. 5). Specific saturated and mono-unsaturated phospholipids, specifically PC 30:0, PC 32:1, and PE 34:1, were identified as downregulated in ER (+) samples and selected as ER profiles.

**Fig. 6:**
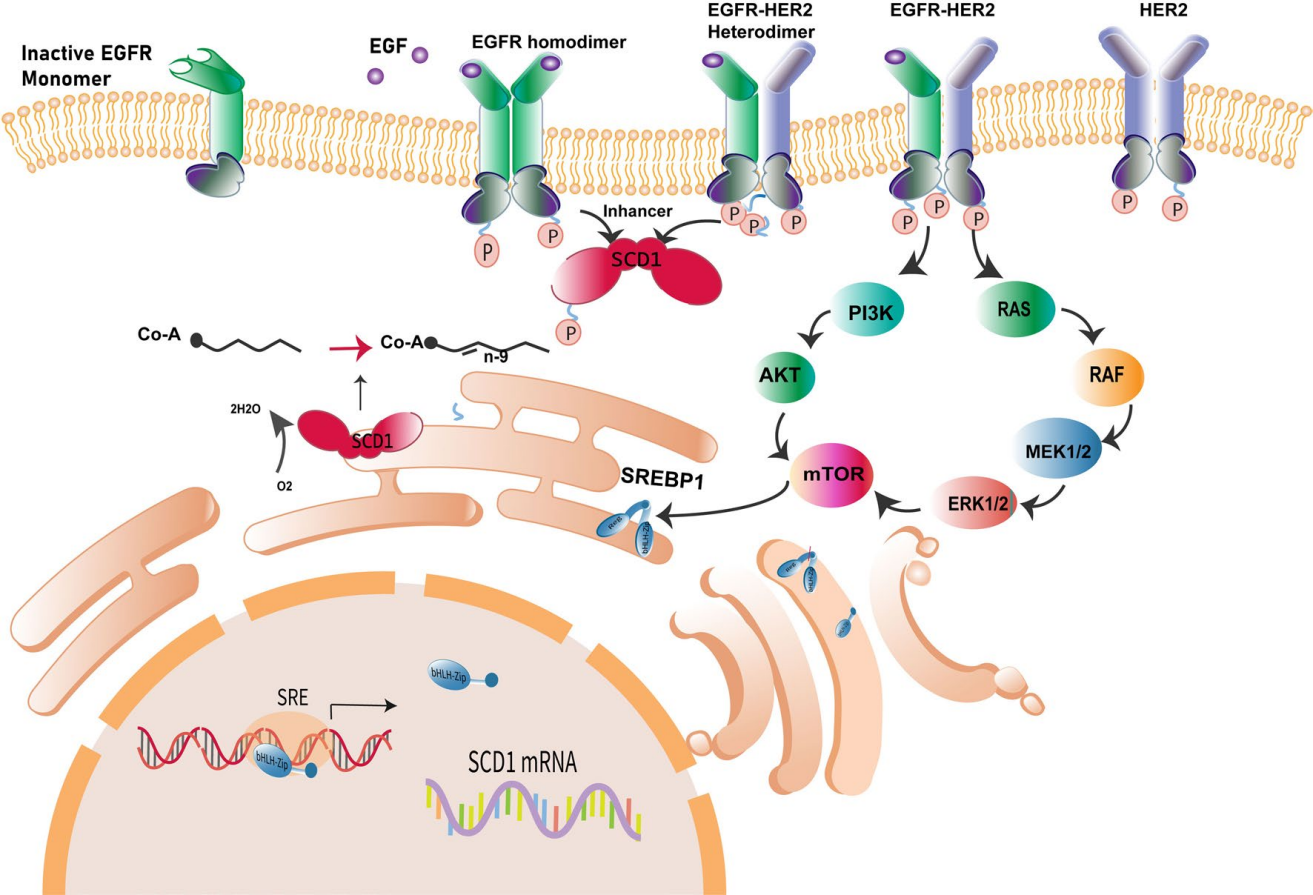
Schematic Representation of Up-Regulation of saturated and mono-unsaturated fatty acids in HER2-Positive Breast Cancer tissues. The EGF family molecules activate an EGFR/HER2 homodimer or an EGFR heterodimer (e.g., EGFR-HER2 receptors). The dimerized EGFR/HER2 complex induces autophosphorylation of tyrosine residues in the carboxyl-terminal, consequently leading to the phosphorylation and activation of SCD1. Additionally, tyrosine phosphorylation triggers a cascade of reactions that activate mTOR (mechanistic target of rapamycin), which in turn regulates the activation of SREBP1 (sterol regulatory element-binding protein 1). SREBP1 is a transcription factor that regulates the expression of SCD1, thereby influencing the production of monounsaturated fatty acids. These fatty acids are incorporated into phospholipids like phosphocholine and phosphoethanolamine, which are crucial for membrane structure and signaling in cancer cells. Initially, SREBP-1 is present as pre-SREBP1 in the endoplasmic reticulum (ER). In the Golgi apparatus, pre-SREBP-1 undergoes two sequential proteolytic cleavages mediated by Site-1 protease (S1P) and Site-2 protease (S2P). These cleavages release the N-terminal fragment of SREBP-1 (nSREBP-1), which contains the basic helix-loop-helix-leucine zipper (bHLH-Zip) domain responsible for DNA binding and transcriptional activation as it binds to the sterol regulatory element-1 (SRE1).

Similarly, only PC 30:0 was selected for the PR status due to its downregulation. At the same time, the HER2 subtype displayed a notable relative overexpression of PC 30:0, PC 30:1, and PC 32:1. This distinct pattern sets it apart from the observed trends in ER (+) and PR (+) samples (Fig. 5B). This finding can be attributed to the involvement of SCD1, a membrane protein in the endoplasmic reticulum responsible for converting saturated and mono-unsaturated phospholipids. SCD1 is highly expressed in breast cancer patients, particularly those with triple-negative and HER2-positive cells, where estrogen receptor and progesterone receptor expression are negative. SCD1 facilitates the conversion of more saturated phospholipids into monounsaturated forms, leading to the overexpression of both saturated and monounsaturated lipids. Conversely, positive estrogen receptor expression acts as a repressor of SCD1, contributing to the decrease in PC 30:0 and PE 32:1 expression in ER (+) tumor tissues, as observed in this study. Figure 6 schematically illustrates how HER2 homo/heterodimers trigger SCD1 overexpression in HER2-positive samples. EGF family molecule activation initiates EGFR homodimer or EGFR-HER2 heterodimer formation, leading to tyrosine phosphorylation at the carboxyl-terminal and subsequent activation of SCD1. The phosphorylation of HER2-HER2/EGFR-HER2 dimer triggers a cascade involving Ras, RAF, MEK1/2, ERK 1/2, PI3k, and AKT, culminating in mTOR activation. mTOR regulates SREBP1 activation, which controls SCD1 expression and influences monounsaturated fatty acid synthesis. Initially, SREBP-1 is pre-SREBP1 in the Endoplasmic Reticulum (ER). In the Golgi apparatus, proteolytic cleavage liberates the N-terminal fragment of SREBP-1 (nSREBP-1), containing the bHLH-Zip domain crucial for DNA binding and transcriptional activation. This fragment binds to sterol regulatory element-1 (SRE1), promoting active transcription of SCD1 mRNA. The resulting increase in SCD1 protein levels elevates saturated and mono-unsaturated fatty acid levels in the cell which incorporated into phospholipids, thereby elucidating the complex regulation of lipid metabolism and signaling pathways in HER2-positive breast cancer.

The reduction in triacylglycerol (TAG) content within specific lipids in breast cancer can be attributed to various factors, including alterations in lipid metabolism, increased lipolysis, fluctuations in the tumor microenvironment, and heightened energy consumption in breast tumor cells^33–37^. Despite the overall decrease in TAG levels, certain TAG species, particularly those containing polyunsaturated fatty acids, are elevated in breast cancer tissues. This selective increase may be driven by the need for specific fatty acids, enhanced lipogenesis and lipid storage (lipid droplet accumulation), and interactions between adipocytes and cancer cells, where adipocytes transfer fatty acids to cancer cells for incorporation into TAGs^23,35,38^.

This study has several caveats and notable limitations, particularly concerning the origin of the normal tissue samples. These samples were obtained from areas surrounding the tumor cells, which may introduce differences in lipid profiles compared to those from a healthy breast. Additionally, it is essential to acknowledge the possibility of errors when manually classifying the presence or absence criteria for ER, PR, and HER2, as this process is highly subjective and reliant on the expertise of the pathologist, potentially leading to variations in interpretations. Moreover, the perfect sensitivity and specificity observed in both the original and external datasets warrant further attention due to dataset imbalance, where the minor group consisted of normal tissue samples. This imbalance led to perfect segregation of cancerous samples, resulting in no false negatives and a sensitivity of 1. While other evaluation metrics also indicate strong performance, perfect specificity raises concerns about overfitting. Overfitting can occur especially if oversampling techniques were used to generate synthetic samples from the small, highly similar normal tissue group, leading the model to learn patterns specific to the training data rather than generalizing to new data. To ensure robustness and generalizability, these results should be validated with additional independent and diverse datasets to confirm the model’s performance and avoid dataset-specific biases.

Nevertheless, the ML-aided workflow demonstrated its functionality for detecting a lipidomics signature in breast cancer. Further investigations using this approach with different datasets and diverse cancer types are necessary to verify the findings proposed in this manuscript. Thus, the sole available lipidomics dataset in breast tissues was employed for external validation. The external validation data demonstrated a similar trend of increasing levels of saturated and mono-unsaturated phospholipids, along with a decrease in TAG content within breast tumor tissues. However, only phosphatidylcholine species, such as PC (32:1), PC (36:1), PC (37:1), and PC (38:1), were consistent with the original dataset. Remarkably, the observed pattern of overexpressed lipids containing Palmitic acid (C16:0), Oleic acid (C18:1), Palmitoleic acid (C16:1), and Stearic acid (C18:0) mirrored the trends observed in saturated and mono-unsaturated phospholipid levels in the original breast cancer samples. The discrepancy between the original and external validity datasets could be attributed to several factors. Firstly, the original dataset contained samples from various breast cancer subtypes, including Luminal A, Luminal B, HER2-enriched, and Triple Negative breast cancer, in addition to non-cancerous samples. In contrast, the validation dataset exclusively comprised TNBC and non-cancerous tissues. TNBC is known to exhibit distinct pathways and lipid metabolism compared to other molecular subtypes^39,40^.

This study systematically employed advanced statistical and machine learning techniques to identify significant lipid profiles, with a particular focus on saturated and monounsaturated phospholipids in breast cancer. The methodology encompassed the entire process from initial data input to comprehensive biological interpretation, demonstrating consistency with existing literature and underscoring the robustness of this workflow. The identification of these notable lipid profiles not only reinforces the reliability of our approach but also provides valuable insights into the lipidomic alterations associated with breast cancer. Furthermore, the versatility of this methodology enables its application across various metabolomics platforms and other omics studies, thereby offering a powerful tool for broader biological and clinical research.

## Materials and methods

### Study population and histopathological examination

The dataset used in this study consisted of LC–MS data on breast tumor samples from the METACancer FP7 project. Detailed sample collection and ethical approval have been previously reported by Hilvo et al.^41^ and Denkert et al.^42^. The breast cancer samples were classified based on HER2, ER, and PR status (Table S1). Additionally, there were 182 PR-positive and 90 PR-negative samples. Tumor histology and grade were evaluated at the primary diagnosis site, with relevant information extracted from the histopathological report. This included the status of the Human Epidermal Growth Factor Receptor 2 (HER2) and the presence or absence of estrogen and progesterone receptors. The number of annotated lipids in LC-positive mode was 183, covering all metadata, including cancerous vs. normal tissue and ER, PR, and HER2 subtype class labels.

The validation dataset was obtained from the Metabolomics Workbench under project PR000742, comprising 70 Triple Negative Breast Cancer (TNBC) samples and 48 benign samples^43^.

### Mass spectrometry of breast tumors

The MS data was previously acquired and processed by Hilvo et al.^41^ and Budczies et al.; the final processed data was used in this study^42^. In brief, lipid extracts were analyzed using Waters Q-Tof Premier MS coupled to Acquity UPLC with Electrospray ionization (ESI) in negative and positive modes. The data were processed using MZmine 2 software, and the lipid identification was based on a combination of an internal spectral library and tandem mass spectrometry^41^. The lipidomics results were normalized according to the total protein content, a method based on measuring the total amount of protein in each sample (mg protein/mg tissue), and the lipids were quantified using internal standards^44^. The external data were acquired using a Triple TOF LC–MS instrument, which offers a wide dynamic range; however, its fragmentation capabilities may not be as extensive as other MS systems such as Q-TOF.

### Machine learning data analysis

All statistical and ML analyses were conducted using R version 4.1.2 except the kPCA analysis that was performed in Python 3.6.

#### Data processing, data transforming, and scaling data

Log transformations were employed to address skewed and heteroscedastic datasets and normalize the data distribution^45^. Subsequently, the datasets were median-centered^46^, and “Autoscaling” or “Pareto” scaling was applied for statistical and ML analysis, reducing potential variable variation to enable a more meaningful comparison^46^.

#### Feature selection

##### Feature selection using machine learning approaches

A comparative analysis of four commonly used feature selection techniques using binary logistic regression was performed to determine the most effective approach for selecting and removing irrelevant or redundant features. The Boruta algorithm, an algorithmic wrapper based on the Random Forest model, was employed for the feature selection^47^. An entropy-based method relying on Shannon’s Entropy and information gain was also applied. The Multilayer Perceptron (MLP), a feedforward neural network, was employed to select features by processing information from input to output without incorporating feedback loops. Furthermore, the importance of features in a partial least square discriminant analysis (PLS-DA) model was assessed using the VIP (Variable Importance in Projection) score. The assessment of these methods aimed to determine the optimal technique for feature selection, which would enhance the predictive power and interpretability of the subsequent models.

##### Biological feature selection:Log2 fold change and effect size analysis

*Log2 fold change* The median log2 fold change (log_2_FC) was employed as a biological feature selection criterion for the origin datasets. The fold change was calculated using the following formula:

$$\text{Fold Change}=\text{log}2\left(\frac{Median\, Cancerous}{Median\, Normal } \right),$$

where Median Cancerous represents the median value of the feature in cancerous samples. Median Normal represents the median value of the feature in normal samples.

An acceptance criterion of log2FC greater than 1.8 or less than − 1.5. For hormone receptors, HER2 groups, and the validation dataset comparison, the cutoff was set at a median of |log2FC| ≥ 1.2^48^. It is important to note that this formula differs from the one used in the original manuscript^37^. Due to the normalization of signal intensity by the overall median in the external validation dataset and the unavailability of raw and non-preprocessed data, fold change calculations were excluded from the external validation analysis.

To quantify the relative abundance ratio of lipids between cancerous and normal tissues, use the following formula:

$$\text{Fold Change}= \frac{Total \,Intensity\, cancer}{Total\, Intensity\, Normal},$$

where Total Intensity Cancer is the sum of the intensities of all cancerous samples for all lipids except triacylglycerol (TAG). Total Intensity Normal is the sum of the intensities of all normal samples for all lipids except triacylglycerol (TAG).

To specifically quantify the relative abundance ratio of saturated and mono-unsaturated phospholipids, use the formula:

$$\text{Fold Change}= \frac{Total\, Intensity\, of\, Saturated \,and \,Mono-unsaturated\, Phospholipids\, in \,Cancer }{Total\, Intensity \,of \,Saturated\, and\, Mono-unsaturated \,Phospholipids \,in\, Normal },$$

where Total Intensity of Saturated and Mono-unsaturated Phospholipids in Cancer is the sum of the intensities of all saturated and mono-unsaturated phospholipids in cancer samples. Total Intensity of Saturated and Mono-unsaturated Phospholipids in Normal is the sum of the intensities of all saturated and mono-unsaturated phospholipids in the normal samples.

In the external validation datasets, signal intensity was normalized using the overall median. As a result, the fold change values were altered. Consequently, the fold change criteria were excluded from the analysis, and the effect size was used exclusively.

*Quantifying the magnitude of group differences (effect size)* Cohen’s effect size (d) was used to determine the difference between the means when evaluating different sets of two comparing groups.

$${\text{d }} = \, \left( {{\text{M1 }} - {\text{ M2}}} \right)/{\text{SDpooled,}}$$

where M1 is the mean of the first group, M2 is the mean of the second group, SDpooled is the pooled standard deviation of both groups.

The values of Cohen’s d were calculated for each lipid across the two groups, with a threshold of Cohen’s d ≥ |1| set to distinguish between cancerous and normal samples in both the original and validation datasets. A cutoff of Cohen’s |d| > 0.4 was considered for other groups of interest^49^.

### Machine learning classification algorithms

#### Support vector machine and random forest analysis

The feature-selected data was oversampled and initially divided into training and testing subsets in a 70:30 ratio. Support Vector Machine (SVM) and Random Forest (RF) classification models were then used to differentiate cancerous from normal samples and to characterize cancer subtypes. SVM variations, including SVM-linear, SVM-radial, and SVM-polynomial, encompassing linear and kernel-based SVMs, were employed for the analysis^50^. Grid search cross-validation with L2 regularization was applied to optimize SVM parameters and avoid overfitting^51^. The Random Forest model was fine-tuned by adjusting two key parameters: the “mtry” parameter, which determines the number of features randomly selected as candidates at each split, and the “ntree” parameter, which controls the number of trees grown in the forest. The optimal “mtry” value was chosen by minimizing the “out of bag error” (OOB), while varying “ntree” values were tested to identify the number of trees that yielded the highest accuracy^52^. To assess the classification performances, we employed the standard deviation (STD) as a means of measuring variability.

#### Univariate and multivariate data analysis

To differentiate metabolites across various classification groups, specific lipids underwent thorough analysis, considering their unique characteristics and changes in quantity. An adjusted Student’s t-test using the Benjamini–Hochberg (BH) method and a Mann–Whitney U test (MWU) were conducted, with a p-value threshold of less than 0.01. For normally distributed data, as determined by a Shapiro–Wilk test (p-value ≥ 0.05), a Welch’s t-test was used. The criteria included a median log2 fold change ≥ 1.8, an effect size of Cohen’s d ≥ |1|, and a Matthews correlation coefficient (MCC) above 0.5 for comparing cancerous and normal groups for both original and external validation data. Additionally, to account for multiple comparisons and reduce the likelihood of false positives, the significance level for each lipid test was adjusted using the Bonferroni correction.

Lipids were selected for hormone receptor and HER2 status based on criteria, including a log2FC of at least 1.2, an effect size of |d| ≥ 0.4, and an MCC of at least 0.40. Furthermore, an MWU and a t-test with a p-value < 0.01, along with Bonferroni correction, were applied to identify the most biologically relevant lipids across groups for further analysis.

Principal Component Analysis (PCA) and Partial Least-Squares Discriminant Analysis (PLS-DA) multivariate analysis were conducted on different study groups. Kernel Principal Component Analysis (kPCA), followed by Logistic Regression (LR), was employed for non-linear dimensionality reduction to enhance differentiation between the predefined classes. A grid search was performed using threefold cross-validation to determine the optimal kernel type and gamma hyperparameter for the kPCA-LR model, where the gamma parameter determined the shape of the kernel function and directly influenced the performance of kPCA^53,54^.

### Model validation

The binary classification performance was assessed with tenfold repeated cross-validation (repeated-CV), using performance indices such as accuracy, sensitivity, specificity, and F1 score as determined by analyzing the resulting confusion matrix. Additionally, the performance of the classification algorithms was evaluated to determine the highest-performing approach for each target group. Subsequently, the Area Under the Receiver Operating Characteristic Curve (AUC-ROC) for individual features was computed based on the selected method. To address dataset imbalance, evaluation metrics such as the F1 score (harmonic mean of precision and recall) and MCC were employed^55–57^. The evaluation metrics were computed using the predictions gathered from the test dataset.

The optimal number of principal components (PCs) for each PCA model was determined by assessing their Q^2^ value through internal cross-validation. The amount of information contributed from each PC was evaluated using the cumulative percent variance (CPV), and the number of PCs in each PCA model was adjusted accordingly. The ROC-AUC curve was utilized for PLS-DA, and the AUC values across PC1, PC2, and PC3 were averaged.

### Pathway and enrichment analysis

To illustrate the interactions between compounds, their reactions, and the roles of enzymes and genes, pathway-based networks were created using the Metscape3 plug-in (version 3.1.3) within Cytoscape (version 3.10.0)^30^. MetaMapp (http://metamapp.fiehnlab.ucdavis.edu) tool generated a correlation network for visualization within the Cytoscape. The alterations in saturated and mono-unsaturated phospholipids were visualized using MetaMapp, which efficiently represents mass spectrometry-based metabolomics datasets as network graphs in Cytoscape, highlighting metabolic changes^58^.

The effect size was applied to compare groups in both the original and validation datasets for enrichment analysis.

## Supplementary Information

Supplementary Information.

## Data Availability

The original datasets, including LC–MS in positive and negative modes and metadata, are available on Fighare. The DOI for accessing these datasets is 10.6084/m9.figshare.25514578. Validation datasets were downloaded from Metabolomics Workbench. This data is available at the NIH Common Fund’s National Metabolomics Data Repository (NMDR) website, the Metabolomics Workbench, https://www.metabolomicsworkbench.org, where it has been assigned Project ID PR000742. The data can be accessed directly via it’s Project 10.21228/M8RX01. https://www.metabolomicsworkbench.org/data/DRCCMetadata.php?Mode=Project&ProjectID=PR000742.

## Code availability

The research data and code are accessible on CodeOcean. The Compute Capsule’s provisional DOI for the code is 10.24433/CO.5011163.v1.
