# Author Correction: Leveraging ML for profiling lipidomic alterations in breast cancer tissues: a methodological perspective

**DOI:** 10.1038/s41598-024-79649-9

**Published:** 2024-12-12

**Authors:** Parisa Shahnazari, Kaveh Kavousi, Zarrin Minuchehr, Bahram Goliaei, Reza M. Salek

**Affiliations:** 1 Laboratory of Complex Biological Systems and Bioinformatics (CBB), Department of Bioinformatics, Institute of Biochemistry and Biophysics (IBB), University of Tehran, Tehran, Iran; 2 Bioinformatics Group, Kish International Campus, University of Tehran, Kish Island, Iran; 3 Department of Systems Biotechnology, National Institute of Genetic Engineering and Biotechnology, Tehran, Iran; 4 Laboratory of Biophysics and Molecular Biology, Institute of Biochemistry and Biophysics (IBB), University of Tehran, Tehran, Iran; 5 School of Clinical Medicine, University of Cambridge, Cambridge Biomedical Campus, Cambridge, CB2 0SP UK

Correction to: *Scientific Reports* 10.1038/s41598-024-71439-7, published online 28 October 2024

The original version of this Article contained errors in the Figures. Figure 4 was published as Figure 5 and Figure 5 was published as Figure 6. Figure 6 was incorrect. The Figure legends were correct at the time of publication.

The original Figures 4, 5 and 6 and accompanying legends appear below.

**Fig. 4:**
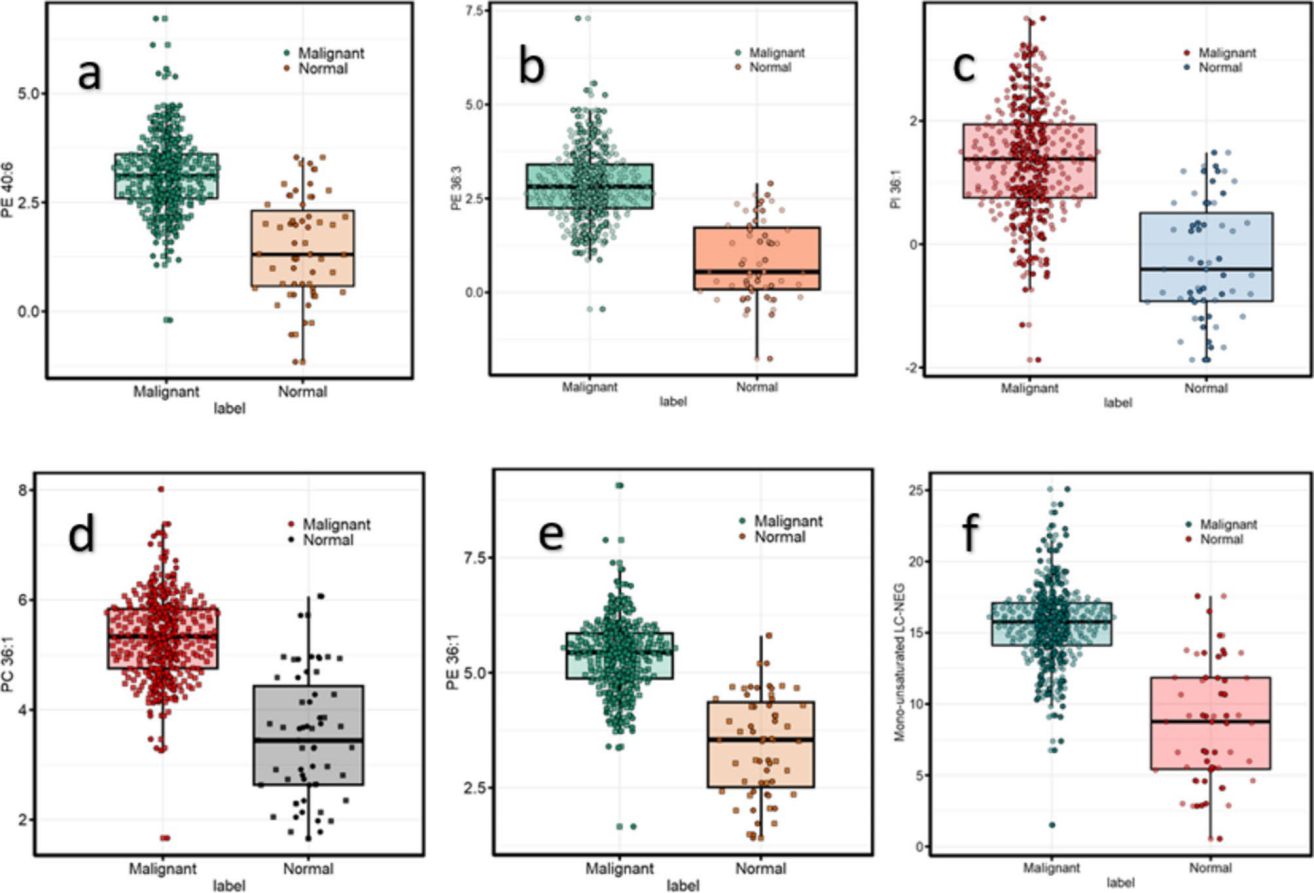
Differential abundance analysis of selected saturated and mono-unsaturated phospholipids based on ER, HER2, and PR Status. (**a**) Downregulation of PC 30:0 and PE 32:1 in ER+ samples. (**b**) Upregulation of PC 30:0 and PC 32:1 in HER2( +) samples. (**c**) Downregulation of PC 30:0 in PR-positive (PR +) breast cancer tissues. For lipids with non-normal distribution, Welch’s correction was excluded. Normality was determined using the Shapiro–Wilk test (p-value < 0.05). A Bonferroni correction was applied to adjust the significance levels to 5.46e−05, 0.00125, and 1.88e−05 for ER, HER2, and PR subtypes, respectively.

**Fig. 5:**
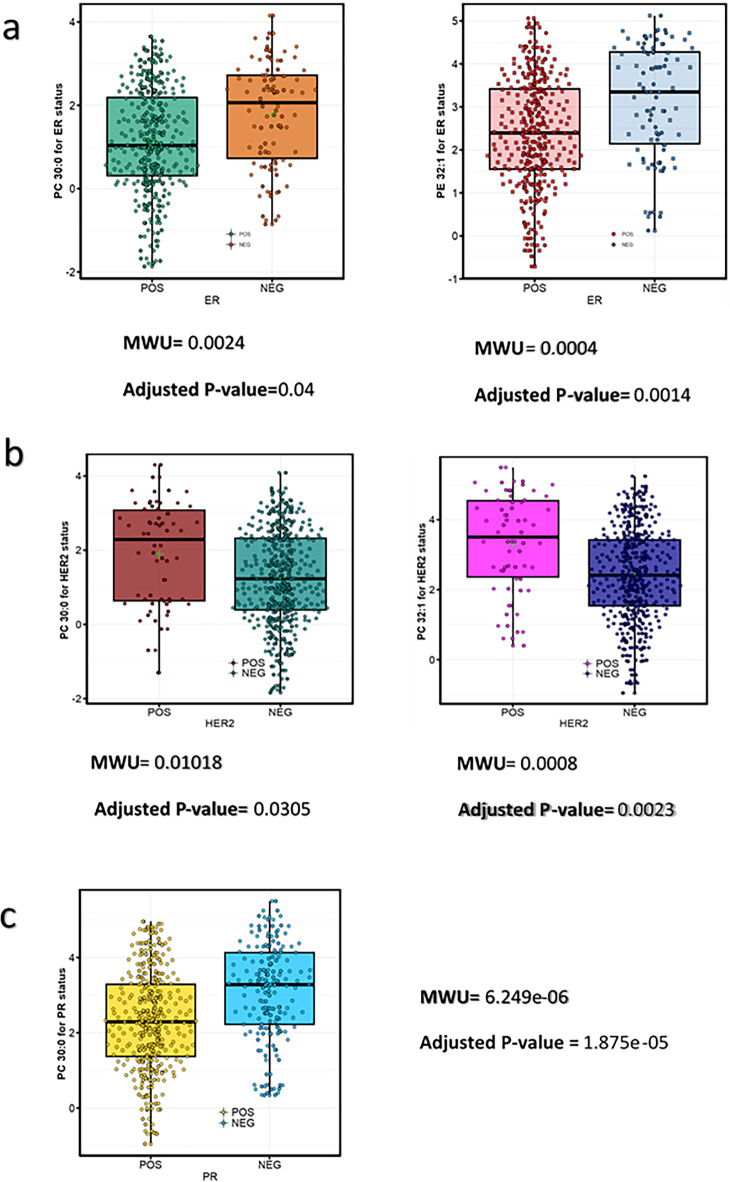
Differential abundance analysis of triacylglycerols (TAGs). (**a**) Differential analysis of TAGs (TAG_42_1, TAG_44_2, TAG_46_2, TAG_48_3, TAG_49_1, TAG_50_4, TAG_50_5, TAG_51_1, TAG_51_2, TAG_52_5, TAG_53_4, TAG_54_6, and TAG_54_7) between cancer and normal samples in positive mode, computed using the defined criteria (MWU and Welch tests). (**b**) PLS-DA score plots with average AUC from PC1, PC2, and PC3. (**c**) Linear-kernel PCA between normal and breast cancer samples. Logistic regression indicates complete segregation between the groups without misclassification.

**Fig. 6:**
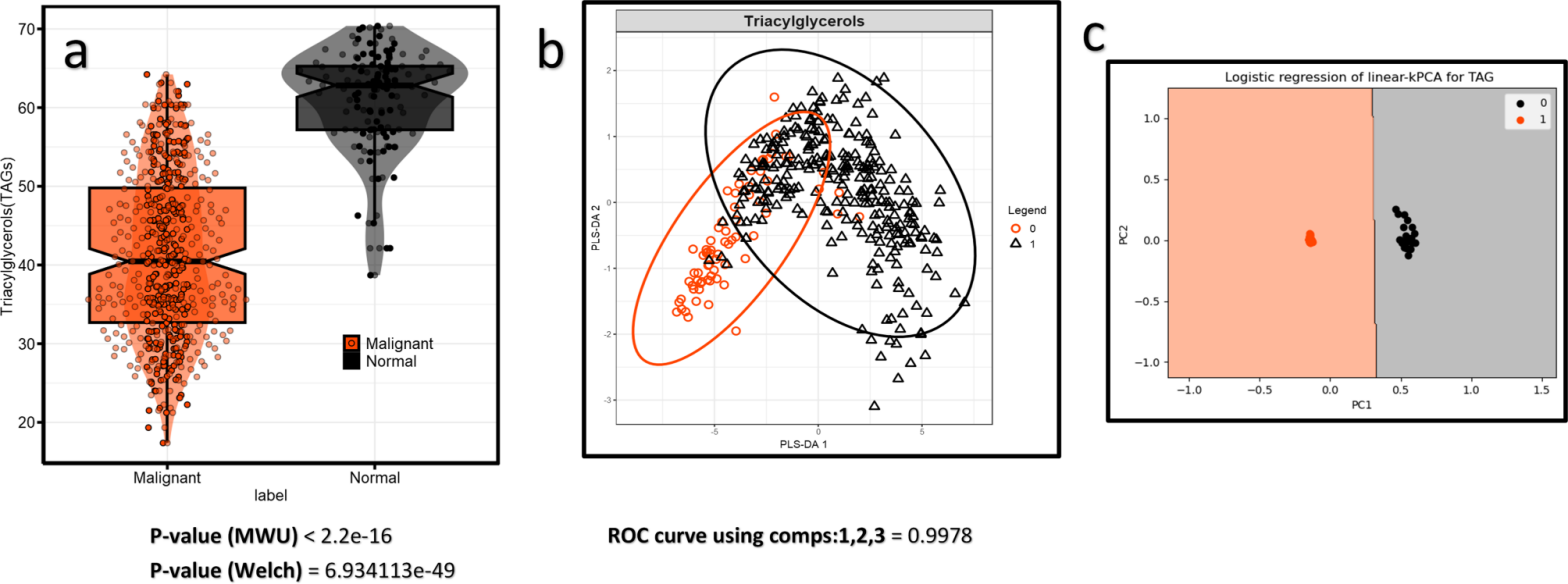
Schematic Representation of Up-Regulation of saturated and mono-unsaturated fatty acids in HER2-Positive Breast Cancer tissues. The EGF family molecules activate an EGFR/HER2 homodimer or an EGFR heterodimer (e.g., EGFR-HER2 receptors). The dimerized EGFR/HER2 complex induces autophosphorylation of tyrosine residues in the carboxyl-terminal, consequently leading to the phosphorylation and activation of SCD1. Additionally, tyrosine phosphorylation triggers a cascade of reactions that activate mTOR (mechanistic target of rapamycin), which in turn regulates the activation of SREBP1 (sterol regulatory element-binding protein 1). SREBP1 is a transcription factor that regulates the expression of SCD1, thereby influencing the production of monounsaturated fatty acids. These fatty acids are incorporated into phospholipids like phosphocholine and phosphoethanolamine, which are crucial for membrane structure and signaling in cancer cells. Initially, SREBP-1 is present as pre-SREBP1 in the endoplasmic reticulum (ER). In the Golgi apparatus, pre-SREBP-1 undergoes two sequential proteolytic cleavages mediated by Site-1 protease (S1P) and Site-2 protease (S2P). These cleavages release the N-terminal fragment of SREBP-1 (nSREBP-1), which contains the basic helix-loop-helix-leucine zipper (bHLH-Zip) domain responsible for DNA binding and transcriptional activation as it binds to the sterol regulatory element-1 (SRE1).

The original Article has been corrected.

