# Supplementary material for: Leveraging ML for profiling lipidomic alterations in breast cancer tissues: a methodological perspective
Source: Sci Rep. 2024 Oct 28;14:25825. doi: 10.1038/s41598-024-71439-7 (PMC11519355; doi:10.1038/s41598-024-71439-7)
Supplement: Supplementary file 1 — Supplementary Information. [file 41598_2024_71439_MOESM1_ESM.docx]

# **Supplement** (Leveraging ML for Profiling Lipidomic Alterations in Breast Cancer Tissues: A Methodological Perspective)

## **Figures:**


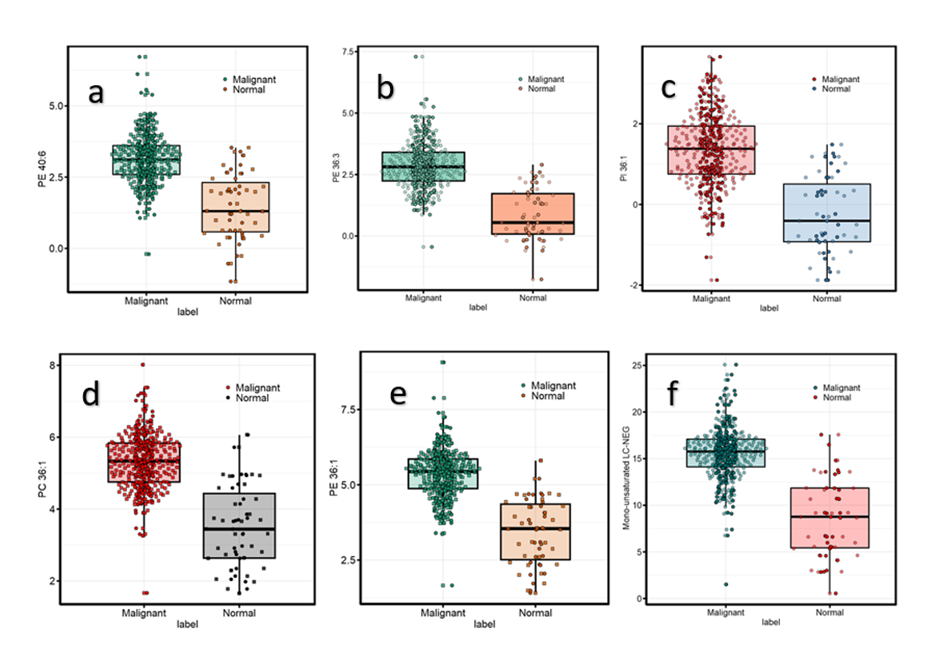


**Fig.S1.** The differential accumulation of selected LC-MS negative mode lipids between normal and malignant tissues is depicted in panels a-f. All significant lipids were identified with a Bonferroni correction applied, resulting in a significance threshold of p < 0.0003. The upregulated phospholipids in cancer cells in the negative mode of LC-MS included PE 40:6, PE 36:3, PE 36:1, PI 36:1, and PC 36:1.

**Fig.S2.** Analysis of Saturated and Mono-unsaturated Compounds Using LC-MS in Positive Mode for External Validation. The lipid species analyzed include LysoPC(15:0), LysoPC(17:1), LysoPC(21:0), LysoPC(24:0), PA(35:1), PC(29:1), PC(32:1), PC(33:0), PC(36:1), PC(37:1), PC(38:1), plasmenyl-PC(30:0), and plasmenyl-PC(37:0). **a)** Differential abundance assessment between cancerous and non-cancerous groups. **b)** PCA analysis revealed a CPV of 54.148% for PC1 and 68.776% for PC2. **c)** PLS-DA resulted in an average AUC of 0.9741 across PC1, PC2, and PC3. **d)** Prediction areas associated with maximum distance for PLS-DA. **e)** RBF-kPCA with logistic regression produced an AUC of 0.9285 and an accuracy of 0.94.

Bonferroni correction adjusted the significance level for each feature to 4.651163e-05. The adjusted p-value (Bonferroni) is 1.5178e-21, the Welch test p-value is 2.2e-16, and the MWU test p-value is 2.2e-16, indicating significance. Normal distribution was detected by the Shapiro-Wilk test (p < 0.05).

**Fig.S3.** Analysis of Saturated and Mono-unsaturated Compounds Using LC-MS in Negative Mode for External Validation. The lipid species analyzed include PE, PI, and PS. **a)** The differential abundance between cancerous and non-cancerous groups demonstrated superior performance in the cancerous group, with the MWU test p-value at 2.396e-15, the Welch test p-value at 2.2e-16, the adjusted p-value at 1.9393e-16, and the Bonferroni correction for each feature at 0.0002. Normal distribution was detected by the Shapiro-Wilk test (p < 0.05). **b)** PCA revealed a CPV of 47.553% for PC1 and 64.891% for PC2. **c)** PLS-DA resulted in an average AUC of 0.9497 across PC1, PC2, and PC3. **d)** Prediction areas associated with maximum distance for PLS-DA. **e)** RBF-kPCA with logistic regression yielded an AUC of 0.9773 and an accuracy of 0.97.

**Fig.S4.** Down-regulation of TAGs in the external validation dataset reveals significant differences between cancerous tumors and normal tissues. a) Differential abundance analysis indicates that TAGs are significantly down-regulated in cancerous tissues compared to non-cancerous tissues. b) The PCA plot shows the variance in the dataset, with Principal Component 1 (PC1) accounting for 80.30% and Principal Component 2 (PC2) accounting for 90.30% of the total variance, effectively separating cancerous and non-cancerous samples. c) PLS-DA classification achieves an AUC of 0.9848, indicating high accuracy in distinguishing between the two tissue types. d) PLS-DA analysis on the external validity of TAGs depicts prediction areas based on maximum prediction distance. All p-values from the t-test (Welch, MWU, and adjusted t-test) were less than the Bonferroni correction threshold of 4.651163e-05, indicating statistically significant results.


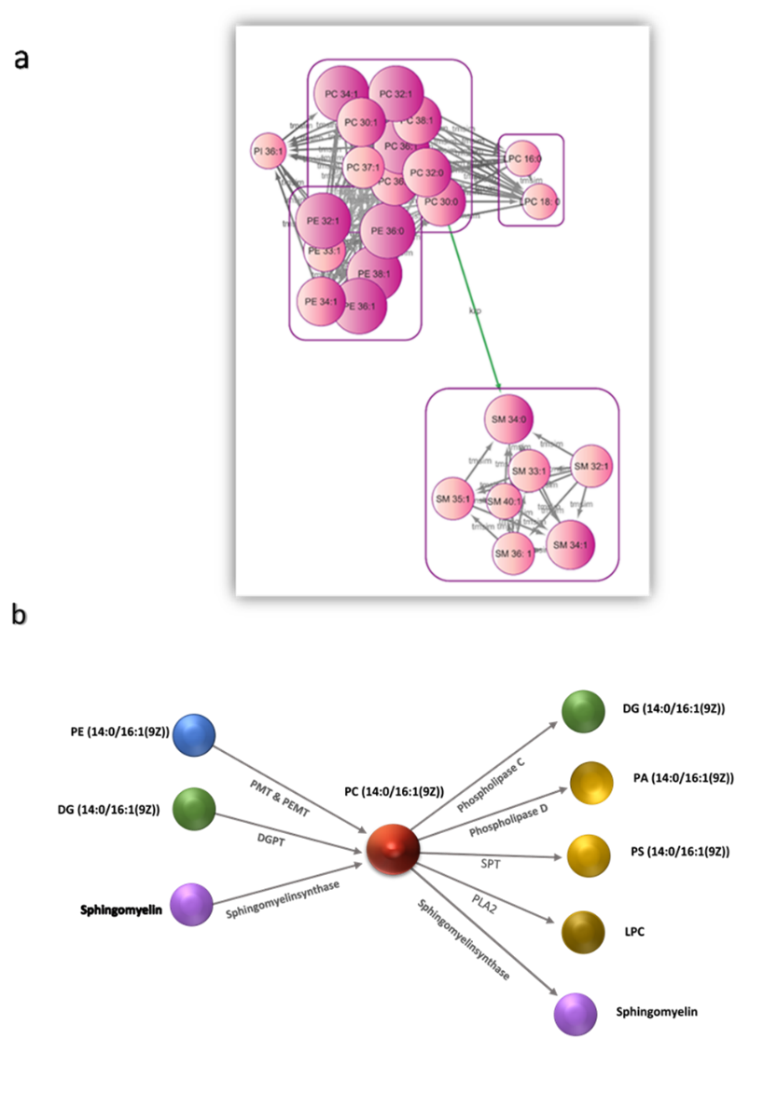


**Fig.S5.** Phospholipid Reactions. a) Interconnection and Compound-Compound Reactions of saturated phospholipids and mono-unsaturated phospholipids (PC, LPC, PE, PI, and SM) using MetaMapp. The nodes in the graph represent metabolites that exhibit increased levels (upregulated) in cancer tissues compared to normal tissues. The size of each node corresponds to the magnitude of the fold-change, ranging from 1.9 to 5.2. The compounds are connected by green lines, representing the KEGG reaction pair (krp), while the gray lines indicate the tanimoto chemical similarity (tmsim) between the metabolites. b) PC (14:0/16:1(9Z))/PC (30:1) conversions occur using specific enzymes to yield DG (30:1), PA (30:1), PS (30:1), LPC, and sphingomyelin. This molecule is a product of reactions involving PE (30:1), DG (30:1), or PC (14:0/0:0) & palmitoleoyl-CoA, catalyzed by the enzymes mentioned below. PMT: Phosphatidyl-N-methylethanolamine N-methyltransferase; PEMT: Phosphatidylethanolamine N-methyltransferase; DGPT: Diacylglycerol cholinephosphotransferase; ABOAT: 1-Acylglycerophosphocholine O-acyltransferase; SPT: L-serine-phosphatidylethanolamine phosphatidyltransferase; PLA2: Phospholipase A(The figure was extracted from Lipidmaps (https://www.lipidmaps.org/databases/lmsd/LMGP01011384).


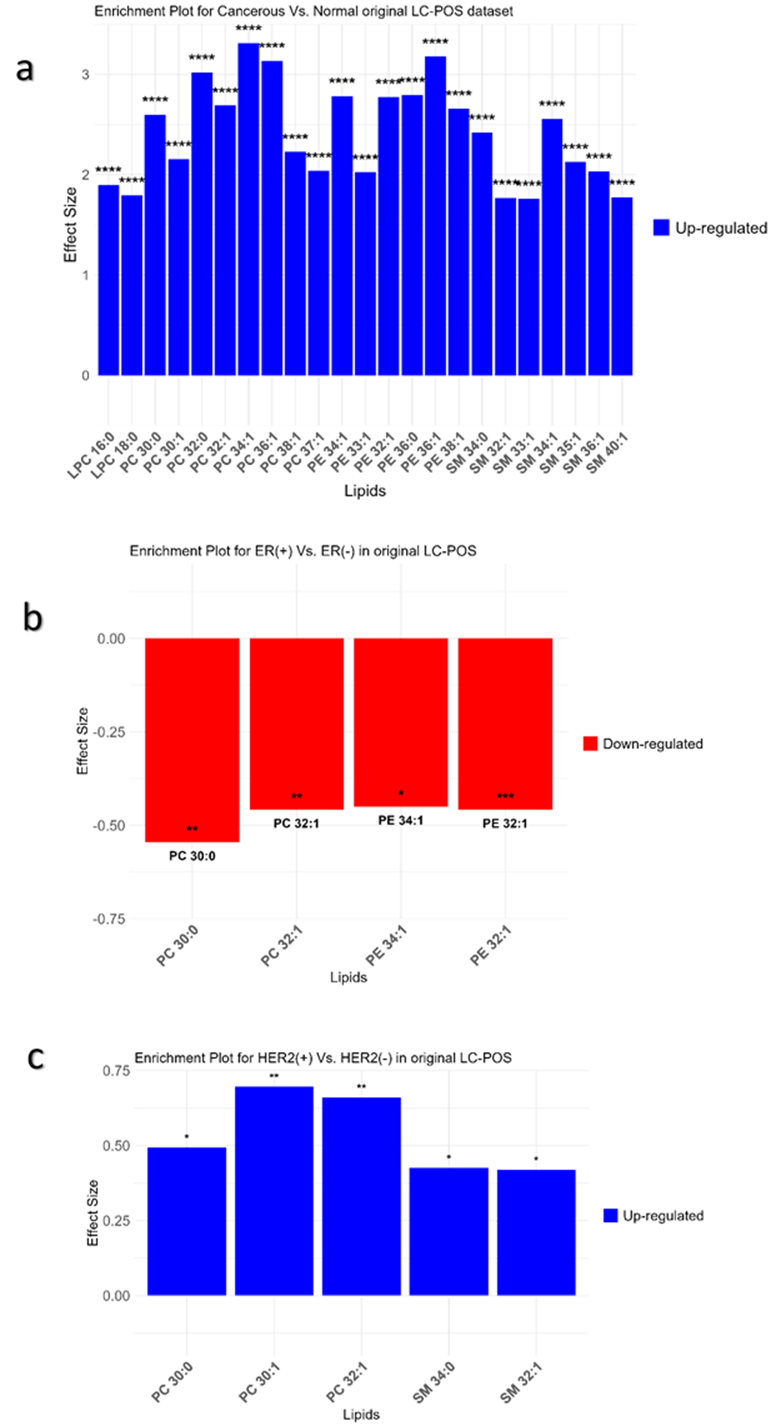


**Fig.S6.** Enrichment plots of saturated and mono-unsaturated phospholipids based on effect size in the positive mode of original datasets. (a) Enrichment plot comparing cancerous versus normal tissues, demonstrating that all selected lipids are up-regulated with notable effect sizes. (b) Enrichment plot comparing ER (+) versus ER (-) tissues, illustrating the down-regulation of all selected phospholipids. (c) Enrichment plot comparing HER2 (+) versus HER2 (-) tissues, illustrating the up-regulation of all selected phospholipids.


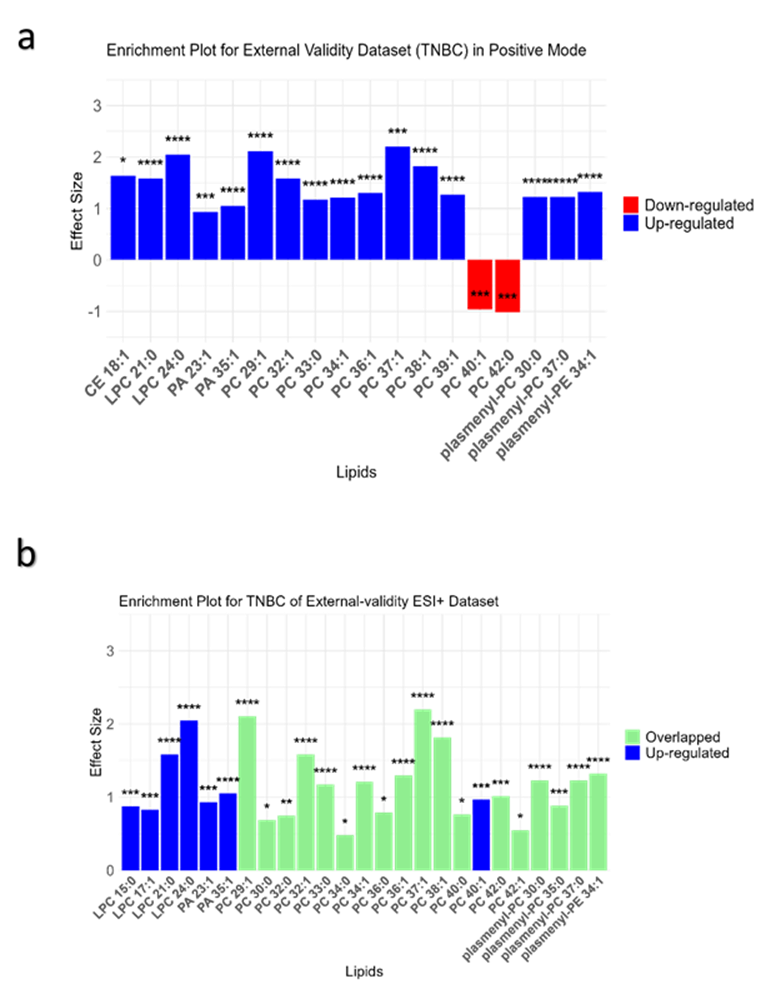


**Fig.S7.** Enrichment plots based on effect size in external validation data in positive mode. **a)** Enrichment plot comparing TNBC versus normal tissues using an external validation dataset. **b**) Enrichment plot illustrating external validity, where light green bars represent saturated and mono-unsaturated phospholipids overlapping with those in the original dataset. Plasmalogens containing structural components such as C16:0 (palmitic acid), C18:0 (stearic acid), C16:1 (palmitoleic acid), and C18:1 (oleic acid) are highlighted as significant overlapping lipids. These components are characteristic of the phospholipids prevalent in breast cancer, reflecting their presence in both the original and validation datasets. Significance levels are indicated as follows: *: p < 0.1, **: p < 0.01, ***: p < 0.001, with Bonferroni correction (4.6512e-05) applied significance level denoted as ****.


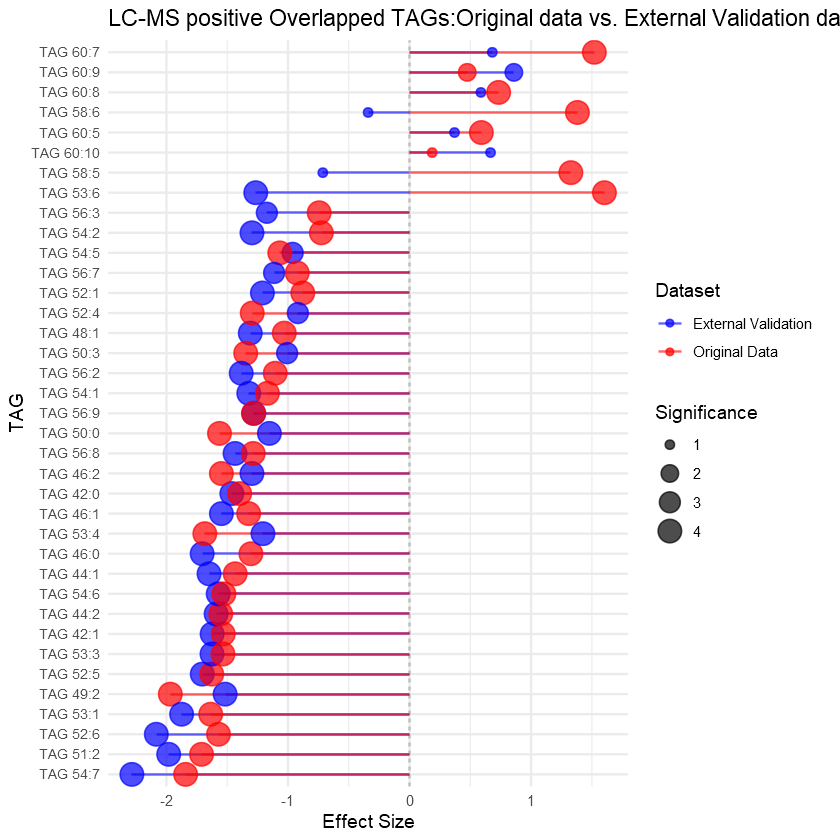

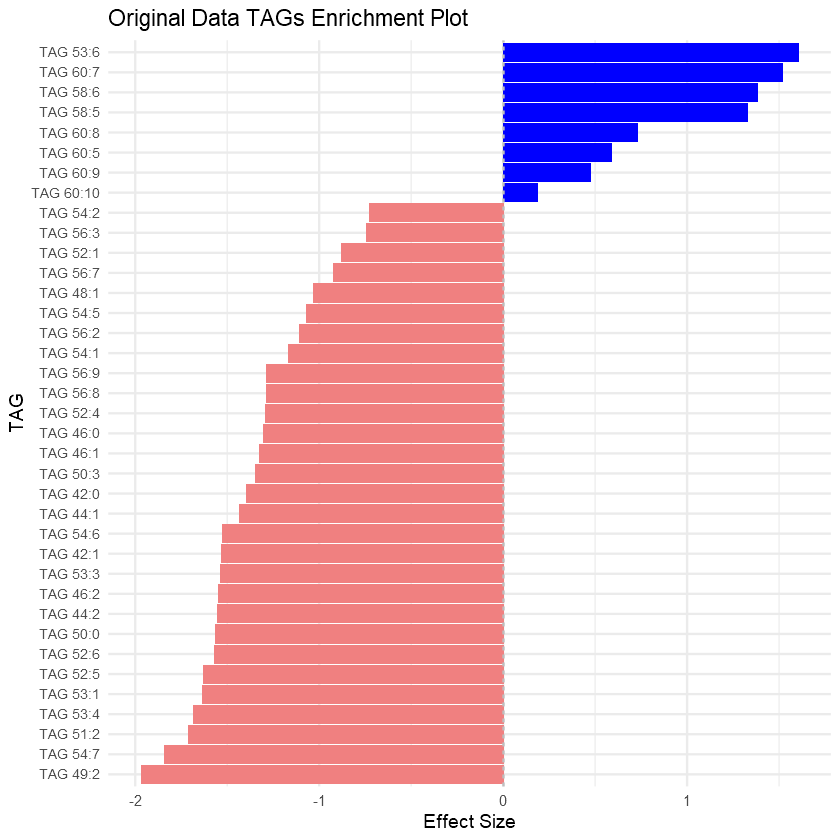

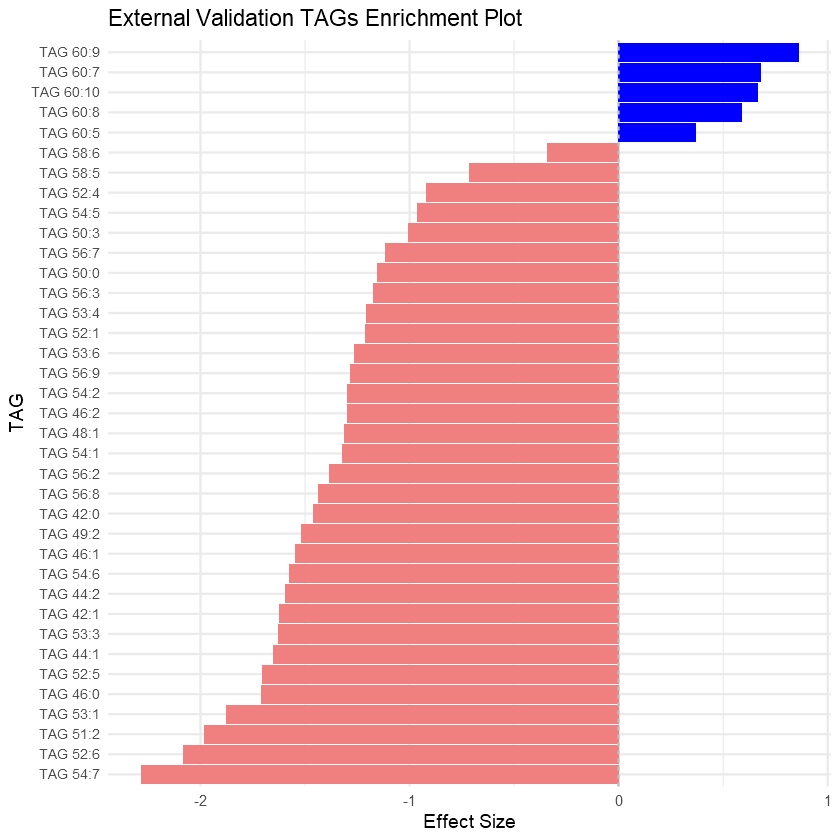


a

b

**Fig.S8. Comparison of TAGs in Original and External Validation Datasets in Positive Mode.** **a)** Enrichment plot comparing TAGs between the original and external validation datasets. **b)** Bubble plot showing the overlap and pattern of effect sizes between the original and external validation TAGs.

## **Tables:**

**Table S1:** Distribution of Samples in Original and External Validation Datasets.

|  | Normal | Cancerous | ER (+) | ER (-) | PR (+) | PR (-) | HER2 (+) | HER2(-) |
| --- | --- | --- | --- | --- | --- | --- | --- | --- |
| Original Data (positive) | 73 | 276 | 67 | 210 | 93 | 184 | 40 | 226 |
| Original Data (Negative) | 31 | 224 | - | - | - | - | - | - |
| External Validation (Pos) | 48 | 70 | - | - | - | - | - | - |
| External Validation (Neg) | 48 | 70 | - | - | - | - | - | - |

**Table S2:** Differential Accumulation of Phospholipids (LPC, PC, PE, PI, and SM) in Positive Mode of LC-MS Between Normal and Cancerous Breast Tissues. All represented lipids have a normal distribution, as determined by the Welch t-test and MWU test with adjusted p-values. Significance levels are indicated by asterisks: *: p < 0.1, **: p < 0.01, ***: p < 0.001. Bonferroni correction (5.464481e-05) applied significance level: ****.

| No | Lipid Name | PubChem-CID | Log2 FC | Adjusted P.value  (t-test) for FC | MWU | Welch | Effect size | Adjusted P.value  for ES |
| --- | --- | --- | --- | --- | --- | --- | --- | --- |
| 1 | Cer 35:1 | 154573063 | 0.7701 | **** | **** | **** | 0.5887 | **** |
| 2 | Cer 42:2 | 154573075 | 2.5715 | **** | **** | **** | 1.8511 | **** |
| 3 | LPC_16_0 | 460602 | 2.5715 | **** | **** | **** | 1.4188 | **** |
| 4 | LPC_18_0 | 497299 | 1.9697 | **** | **** | **** | 1.7305 | **** |
| 5 | PC 30:0 | 129657 | 3.9494 | **** | **** | **** | 2.4668 | **** |
| 6 | PC 30:1 | 24778575 | 3.4431 | **** | **** | **** | 2.0723 | **** |
| 7 | PC 32:0 | 452110 | 4.0752 | **** | **** | **** | 2.8870 | **** |
| 8 | PC 32:1 | 53478711 | 4.5530 | **** | **** | **** | 2.5616 | **** |
| 9 | PC 32:2 | 24778764 | 3.6304 | **** | **** | **** | 2.2218 | **** |
| 10 | PC 32:5 | 52922270 | 3.4678 | **** | **** | **** | 2.3962 | **** |
| 11 | PC 34:1 | 5497103 | 4.1202 | **** | **** | **** | 3.1363 | **** |
| 12 | PC 34:2 | 5287971 | 3.3443 | **** | **** | **** | 2.4390 | **** |
| 13 | PC 34:3 | 53478943 | 3.3991 | **** | **** | **** | 2.4600 | **** |
| 14 | PC 34:4 | 24778706 | 4.4842 | **** | **** | **** | 2.6873 | **** |
| 15 | PC 32:4 | 52922765 | 3.9184 | **** | **** | **** | 2.5268 | **** |
| 16 | PC 36:1 | 24778825 | 4.5387 | **** | **** | **** | 2.9751 | **** |
| 17 | PC 36:2 | 10350317 | 3.5984 | **** | **** | **** | 2.7117 | **** |
| 18 | PC 36:3 | 53478785 | 4.0604 | **** | **** | **** | 2.5910 | **** |
| 19 | PC 36:4 | 52922783 | 3.4874 | **** | **** | **** | 2.7063 | **** |
| 20 | PC 36:5 | 53478611 | 3.1393 | **** | **** | **** | 2.6118 | **** |
| 21 | PC 38:1 | 24778843 | 3.174 | **** | **** | **** | 2.1191 | **** |
| 23 | PC 37:1 | 52922336 | 3.0742 | **** | **** | **** | 1.9688 | **** |
| 24 | PC 38:2 | 24778897 | 4.2486 | **** | **** | **** | 2.6480 | **** |
| 25 | PC 38:3 | 53478735 | 4.7162 | **** | **** | **** | 2.7697 | **** |
| 26 | PC 37:3 | 52922561 | 2.6562 | **** | **** | **** | 2.0319 | **** |
| 27 | PC 38:4 | 16219824 | 3.849 | **** | **** | **** | 2.4127 | **** |
| 28 | PC 38:5 | 52922801 | 3.3546 | **** | **** | **** | 2.7667 | **** |
| 29 | PC 37:5 | 52922402 | 3.5856 | **** | **** | **** | 2.5334 | **** |
| 30 | PC 38:6 | 6441886 | 3.6472 | **** | **** | **** | 2.3507 | **** |
| 31 | PC 38:7 | 24778773 | 4.0497 | **** | **** | **** | 2.6997 | **** |
| 32 | PC 40:4 | 53479183 | 3.6592 | **** | **** | **** | 2.4023 | **** |
| 33 | PC 40:5 | 52922705 | 4.0511 | **** | **** | **** | 2.6167 | **** |
| 34 | PC 40:6 | 52922757 | 3.7568 | **** | **** | **** | 2.3803 | **** |
| 35 | PC 40:7 | 52922817 | 3.8607 | **** | **** | **** | 2.6040 | **** |
| 36 | PC 40:8 | 52922937 | 1.7056 | **** | **** | **** | 1.2958 | **** |
| 37 | PC 40:3 | 24779047 | 2.3051 | **** | **** | **** | 1.9185 | **** |
| 38 | PC 40:9 | 52923373 | 3.208 | **** | **** | **** | 1.9748 | **** |
| 39 | PE 34:0 | 46891875 | 0.3991 | **** | **** | **** | 0.4703 | **** |
| 40 | PE 34:1 | 5283496 | 4.0335 | **** | **** | **** | 2.6589 | **** |
| 41 | PE 33:1 | 52924162 | 3.9920 | **** | **** | **** | 2.5690 | **** |
| 42 | PE 34:2 | 52924121 | 3.7089 | **** | **** | **** | 2.3430 | **** |
| 43 | PE 33:2 | 52924189 | 4.332 | **** | **** | **** | 2.8726 | **** |
| 44 | PE 31:5 | 134728900 | 1.3416 | **** | **** | **** | 1.4483 | **** |
| 45 | PE 32:1 | 52994944 | 5.2049 | **** | **** | **** | 2.6918 | **** |
| 46 | PE 36:0 | 52924864 | 4.8063 | **** | **** | **** | 2.9237 | **** |
| 47 | PE 36:1 | 9546755 | 4.6686 | **** | **** | **** | 3.0144 | **** |
| 48 | PE 36:2 | 6437392 | 3.9922 | **** | **** | **** | 2.7264 | **** |
| 49 | PE 36:3 | 52924364 | 1.8095 | **** | **** | **** | 1.4991 | **** |
| 50 | PE 34:3 | 52924147 | 2.6765 | **** | **** | **** | 1.6204 | **** |
| 51 | PE 35:3 | 52924303 | 2.8146 | **** | **** | **** | 1.9284 | **** |
| 52 | PE 36:4 | 52924875 | 3.2235 | **** | **** | **** | 1.9143 | **** |
| 53 | PE 38:1 | 9546837 | 4.2005 | **** | **** | **** | 2.5499 | **** |
| 54 | PE 38:2 | 52924239 | 4.2969 | **** | **** | **** | 2.4851 | **** |
| 55 | PE 38:3 | 52924240 | 3.3397 | **** | **** | **** | 2.2313 | **** |
| 56 | PE 38:4 | 5289133 | 4.2434 | **** | **** | **** | 2.9908 | **** |
| 57 | PE 38:5 | 52924373 | 2.9856 | **** | **** | **** | 2.2723 | **** |
| 58 | PE 37:5 | 52924808 | 2.8798 | **** | **** | **** | 2.2179 | **** |
| 59 | PE 37:6 | 52924672 | 3.8066 | **** | **** | **** | 2.5464 | **** |
| 60 | PE 37:7 | 85332412 | 4.6664 | **** | **** | **** | 2.7158 | **** |
| 61 | PE 39:0 | 52924482 | -2.1448 | **** | **** | **** | -1.4260 | **** |
| 62 | PE 40:4 | 52924901 | 3.5982 | **** | **** | **** | 2.4291 | **** |
| 63 | PE 40:6 | 9546798 | 2.9708 | **** | **** | **** | 2.1415 | **** |
| 64 | PE 39:6 | 52924320 | 1.2641 | **** | **** | **** | 0.9577 | **** |
| 65 | SM 34:0 | 9939965 | 3.5477 | **** | **** | **** | 2.3057 | **** |
| 66 | SM 32:1 | 11433862 | 2.4452 | **** | **** | **** | 1.6887 | **** |
| 67 | SM 33:1 | 52931137 | 2.5254 | **** | **** | **** | 1.6754 | **** |
| 68 | SM 33:2 | 164465718 | 0.2049 | * | * | * | 0.0594 | * |
| 69 | SM 34:1 | 9939941 | 3.5546 | **** | **** | **** | 2.3903 | **** |
| 70 | SM 35:1 | 46891763 | 3.0221 | **** | **** | **** | 2.0279 | **** |
| 71 | SM 36:1 | 52931165 | 2.7056 | **** | **** | **** | 1.9221 | **** |
| 72 | SM 36:4 | 164504937 | 4.2845 | **** | **** | **** | 2.5685 | **** |
| 73 | SM 37:2 | 164388636 | 0.113 | ** | * | *** | 0.2935 | ** |
| 74 | SM 37:3 | 164462061 | 1.0849 | **** | **** | **** | 1.1414 | **** |
| 75 | SM 40:1 | 52931165 | 2.0835 | **** | **** | **** | 1.6937 | **** |
| 76 | SM 39:1 | 154573100 | 0.564 | **** | **** | **** | 0.7562 | **** |
| 77 | SM 39:2 | 154573490 | 3.2609 | **** | **** | **** | 2.2067 | **** |
| 78 | SM 42:2 | 154573110 | 3.2376 | **** | **** | **** | 2.0803 | **** |

**Table S3**: Differential Accumulation of Phospholipids in Original Data in Negative Mode Between Cancerous and Normal Tissues. The Welch t-test was used, excluding data with non-normal distributions (NA). Significance levels are indicated by asterisks: *: p < 0.1, **: p < 0.01, ***: p < 0.001. Bonferroni correction (0.0003) applied significance level: ****.

| No |  | PubChem-CID | Log2 FC | Adjusted P.value  (t-test) for FC | MWU | Welch | Effect size | Adjusted P.value for ES |
| --- | --- | --- | --- | --- | --- | --- | --- | --- |
| 1 | PC 32:0 | 452110 | 0.8736 | **** | **** | NA | 1.3262 | **** |
| 2 | PC 34:1 | 5497103 | 1.683 | **** | **** | NA | 1.9386 | **** |
| 3 | PC 34:2 | 5287971 | 0.9304 | ** | *** | NA | 0.9046 | * |
| 4 | PC 36:3 | 53478785 | 1.3147 | **** | **** | NA | 1.5211 | **** |
| 5 | PC36:4 | 52922783 | 0.8919 | *** | **** | NA | 1.1017 | ** |
| 6 | PE 38:4 | 16219824 | 1.4716 | **** | **** | NA | 1.8071 | **** |
| 7 | PC 38:6 | 6441886 | 0.9291 | ** | *** | NA | 0.8912 | * |
| 8 | PC 32:1 | 53478711 | 2.1875 | **** | **** | NA | 1.5124 | **** |
| 9 | PEO 40:6 | 160713633 |  | * | ** | NA | 0.5810 | * |
| 10 | PC 38:3 | 53478735 | 1.3187 | **** | **** | NA | 1.4472 | **** |
| 11 | PC 38:4 | 16219824 | 0.8832 | **** | **** | NA | 1.3477 | **** |
| 12 | PC 36:1 | 24778825 | 1.9309 | **** | **** | NA | 2.1349 | **** |
| 13 | PC 38:5 | 52922801 | 0.0942 | * | * | NA | 0.0874 | * |
| 14 | PE 36:2 | 6437392 | 1.6857 | **** | **** | NA | 1.8764 | **** |
| 15 | PE 38:2 | 52924239 | 0.7945 | ** | **** | NA | 1.0598 | ** |
| 16 | PC 36:2 | 24778764 | 0.8343 | ** | **** | NA | 1.0929 | ** |
| 17 | PE 36:1 | 9546734 | 2.033 | **** | **** | NA | 2.2248 | **** |
| 18 | PE 40:6 | 134729615 | 1.9477 | **** | **** | NA | 1.8239 | **** |
| 19 | PE 35:1 | 52924108 | 1.8566 | **** | **** | NA | 1.3884 | **** |
| 20 | PE 36:3 | 52924364 | 2.7186 | **** | **** | NA | 2.0016 | **** |
| 21 | PCO 32:0 | 252150170 | 1.3847 | **** | **** | NA | 1.4894 | **** |
| 22 | PEO 37:5 | 164504490 | 0.6356 | * | *** | NA | 0.8809 | * |
| 23 | PEO 34:2 | 6443157 | 2.0594 | **** | **** | NA | 1.8909 | **** |
| 24 | PEO 34:3 | 53481709 | 1.8779 | **** | **** | NA | 1.6757 | **** |
| 25 | PE 35:5 | 164337328 | 1.0596 | *** | **** | NA | 1.2700 | ** |
| 26 | PEO 36:2 | 164337328 | 1.3210 | ** | *** | NA | 1.0115 | * |
| 27 | PEO 38:5 | 24779379 | 0.4223 | * | * | NA | 0.7908 | * |
| 28 | PI 36:1 | 52927527 | 1.9069 | **** | **** | NA | 1.7397 | **** |
| 29 | PI 38:3 | 138159922 | 1.3316 | ** | **** | NA | 1.2386 | *** |
| 30 | PE 40:7 | 52928033 | 1.2056 | **** | **** | NA | 1.3602 | **** |
| 31 | SM 40:1 | 154573102 | 0.2652 | * | * | NA | 0.0145 | * |
| 32 | SM 34:1 | 154573090 | 1.0168 | ** | **** | NA | 1.1079 | **** |
| 33 | SM 31:2 | 164204914 | 1.0597 | ** | **** | NA | 1.0643 | ** |
| 34 | SM 41:2 | 154573182 | 0.8345 | * | *** | NA | 0.8849 | * |
| 35 | SM 42:2 | 154573110 | 0.5806 | * | *** | NA | 0.6212 | * |
| 36 | SM 43:2 | 154573186 | 0.0095 | * | * | NA | -0.0070 | * |

**Table S4:** Table S4: Differential Accumulation of Triacylglycerol (TAG) in Original Data in Positive Mode Between Cancerous and Normal Tissues. The Welch t-test was used, excluding data with non-normal distributions (NA). Significance levels are indicated by asterisks: *: p < 0.1, **: p < 0.01, ***: p < 0.001. Bonferroni correction (6.060606×10−5) applied significance level: ****.

| No | Lipid Name | PubChem-CID | Log2 FC | Adjusted P.value  (t-test) for FC | MWU | Welch | Effect size | Adjusted P.value  for ES |
| --- | --- | --- | --- | --- | --- | --- | --- | --- |
| 1 | TAG 42:0 | 11148 | -1.3874 | **** | **** | **** | -1.3972 | **** |
| 2 | TAG 42:1 | 56936439 | -2.073 | **** | **** | **** | -1.5325 | **** |
| 3 | TAG 44:0 | 348296890 | -1.0071 | **** | **** | **** | -1.3059 | **** |
| 4 | TAG 44:1 | 348296896 | -1.6122 | **** | **** | **** | -1.4343 | **** |
| 5 | TAG 44:2 | 56936448 | -1.817 | **** | **** | **** | -1.553 | **** |
| 6 | TAG 45:0 | 131753239 | -0.0269 | * | * | NA | -0.2991 | * |
| 7 | TAG 45:1 | 56938362 | -1.3603 | **** | **** | **** | -1.4175 | **** |
| 8 | TAG 45:2 | 131754297 | -1.4704 | **** | **** | **** | -1.2626 | **** |
| 9 | TAG 46:0 | 131754067 | -0.9032 | **** | **** | NA | -1.3051 | **** |
| 10 | TAG 46:1 | 131750529 | -0.9256 | **** | **** | NA | -1.3236 | **** |
| 11 | TAG 46:2 | 56936466 | -1.571 | **** | **** | **** | -1.548 | **** |
| 12 | TAG 47:1 | 131753246 | -1.4338 | **** | **** | **** | -1.5778 | **** |
| 13 | TAG 47:2 | 85332546 | -1.9014 | **** | **** | **** | -1.7305 | **** |
| 14 | TAG 48:0 | 252150192 | -0.6847 | **** | **** | NA | -1.0121 | **** |
| 15 | TAG 48:1 | 9543986 | -0.5153 | **** | **** | NA | -1.0307 | **** |
| 16 | TAG 48:2 | 131750532 | -0.7186 | **** | **** | NA | -1.4322 | **** |
| 17 | TAG 48:3 | 56936516 | -1.684 | **** | **** | NA | -1.560 | **** |
| 18 | TAG 48:4 | 131753431 | -1.369 | **** | **** | **** | -1.2868 | **** |
| 19 | TAG 49:0 | 131754177 | -1.3586 | **** | **** | **** | -1.5118 | **** |
| 20 | TAG 49:1 | 56936678 | -1.755 | **** | **** | NA | - 1.610 | **** |
| 21 | TAG 49:2 | 131753254 | -1.6781 | **** | **** | NA | -1.968 | **** |
| 22 | TAG 49:3 | 131753255 | -0.6664 | * | ** | NA | -0.2089 | * |
| 23 | TAG 50:0 | 3246953 | -1.7322 | **** | **** | NA | -1.5635 | **** |
| 24 | TAG 50:1 | 11308890 | -0.1636 | **** | **** | NA | -0.6847 | **** |
| 25 | TAG 50:3 | 405238211 | -0.5879 | **** | **** | NA | -1.3479 | **** |
| 26 | TAG 50:4 | 25240359 | -1.802 | **** | **** | **** | -1.651 | **** |
| 27 | TAG 50:5 | 56937262 | -1.848 | **** | **** | **** | -2.414 | **** |
| 28 | TAG 51:1 | 56936775 | -2.04 | **** | **** | NA | -1.409 | **** |
| 29 | TAG 51:2 | 56936536 | -1.5317 | **** | **** | NA | -1. 7112 | **** |
| 30 | TAG 51:3 | 131750646 | -1.8177 | **** | **** | NA | -2.0792 | **** |
| 31 | TAG 51:4 | 131750647 | -0.8884 | **** | **** | **** | -1.5375 | **** |
| 32 | TAG 52:1 | 25240360 | -0.5861 | **** | **** | **** | -0.8784 | **** |
| 33 | TAG 52:4 | 405234161 | -0.5295 | **** | **** | **** | -1.2942 | **** |
| 34 | TAG 52:5 | 56937304 | -1.764 | **** | **** | **** | -1.627 | **** |
| 35 | TAG 52:6 | 405234891 | -1.3076 | **** | **** | **** | -1.5716 | **** |
| 36 | TAG 53:1 | 131753251 | -1.8225 | **** | **** | **** | -1.6356 | **** |
| 37 | TAG 53:3 | 131754194 | -1.1425 | **** | **** | **** | -1.535 | **** |
| 38 | TAG 53:4 | 56938920 | -1.849 | **** | **** | **** | -1.684 | **** |
| 39 | TAG 53:5 | 131754532 | -0.8081 | **** | **** | **** | -1.0282 | **** |
| 40 | TAG 53:6 | 9544242 | 2.823 | **** | **** | NA | 1.6030 | **** |
| 41 | TAG 54:1 | 56936651 | -2.03 | **** | **** | **** | -1.1694 | **** |
| 42 | TAG 54:2 | 9544199 | -0.3983 | **** | **** | **** | -0.7259 | **** |
| 43 | TAG 54:4 | 25240371 | -0.1914 | **** | **** | **** | -0.6273 | **** |
| 44 | TAG 54:5 | 405236248 | -0.8186 | **** | **** | **** | -1.0674 | **** |
| 45 | TAG 54:6 | 5322095 | -1.727 | **** | **** | **** | -1.527 | **** |
| 46 | TAG 54:7 | 348276688 | -1.913 | **** | **** | **** | -1.842 | **** |
| 47 | TAG 54:8 | 131753764 | -0.7226 | * | **** | NA | -0.5390 | ** |
| 48 | TAG 55:0 | 131754261 | 0.3069 | * | **** | NA | 0.6690 | ** |
| 49 | TAG 55:1 | 131754165 | 0.3399 | * | * | NA | 0.2240 | * |
| 50 | TAG 55:6 | 131754447 | 1.1022 | **** | **** | **** | 1.1019 | **** |
| 51 | TAG 56:2 | 131753370 | -2.1312 | **** | **** | NA | -1.1051 | **** |
| 52 | TAG 56:3 | 9544447 | -0.7085 | **** | **** | **** | -0.7437 | **** |
| 53 | TAG 56:7 | 131750416 | -1.0372 | **** | **** | **** | -0.9243 | **** |
| 54 | TAG 56:8 | 56939941 | -1.705 | **** | **** | **** | -1.286 | **** |
| 55 | TAG 56:9 | 131750406 | -1.3753 | **** | **** | **** | -1.2839 | **** |
| 56 | TAG 57:2 | 1 31754249 | 1.2569 | **** | **** | **** | 1.1900 | **** |
| 57 | TAG 57:5 | 131754621 | 1.4092 | **** | **** | **** | 1.3904 | **** |
| 58 | TAG 58:2 | 131750420 | -1.1536 | ** | **** | NA | -0.8181 | **** |
| 59 | TAG 58:5 | 131750371 | 1.0875 | **** | **** | **** | 1.3269 | **** |
| 60 | TAG 58:6 | 131750413 | 1.2919 | **** | **** | **** | 1.3807 | **** |
| 61 | TAG 58:8 | 131750572 | -0.5490 | * | *** | NA | -0.5673 | * |
| 62 | TAG 60:4 | 131750377 | -0.7437 | * | **** | NA | -0.6065 | * |
| 64 | TAG 60:5 | 131750379 | 0.4661 | **** | **** | NA | 0.5906 | **** |
| 65 | TAG 60:7 | 131756151 | 1.5359 | **** | **** | **** | 1.5199 | **** |
| 66 | TAG 60:8 | 131756157 | 0.5900 | **** | **** | NA | 0.7326 | **** |
| 67 | TAG 60:9 | 131750419 | 0.3566 | ** | ** | NA | 0.4744 | ** |
| 68 | TAG 60:10 | 131750619 | 0.1022 | * | * | NA | 0.1860 | * |
